# Multipath cycleGAN for harmonization of paired and unpaired low‐dose lung computed tomography reconstruction kernels

**DOI:** 10.1002/mp.70120

**Published:** 2025-11-08

**Authors:** Aravind R. Krishnan, Thomas Z. Li, Lucas W. Remedios, Michael E. Kim, Chenyu Gao, Gaurav Rudravaram, Elyssa M. McMaster, Adam M. Saunders, Shunxing Bao, Kaiwen Xu, Lianrui Zuo, Kim L. Sandler, Fabien Maldonado, Yuankai Huo, Bennett A. Landman

**Affiliations:** ^1^ Department of Electrical and Computer Engineering Vanderbilt University Nashville USA; ^2^ Department of Biomedical Engineering Vanderbilt University Nashville USA; ^3^ Department of Computer Science Vanderbilt University Nashville USA; ^4^ Insitro South San Francisco USA; ^5^ Department of Radiology and Radiological Sciences Vanderbilt University Medical Center Nashville USA; ^6^ Department of Medicine Vanderbilt University Medical Center Nashville USA; ^7^ Department of Thoracic Surgery Vanderbilt University Medical Center Nashville USA; ^8^ Vanderbilt University Institute of Imaging Science Vanderbilt University Medical Center Nashville USA

**Keywords:** multipath cycleGAN, percent emphysema, reconstruction kernel, shared latent space

## Abstract

**Background:**

Reconstruction kernels in computed tomography (CT) introduce variability in spatial resolution and noise distribution, creating systematic differences in quantitative imaging measurements, that icl, emphysema characterization in lung imaging. While harmonization across images reconstructed using different kernels from the same manufacturer is feasible, this can be challenging in multi‐centre or longitudinal studies where images are acquired using kernels from different manufacturers. This variability results in heterogeneous quantitative measurements that are difficult to compare. Therefore, it is necessary to standardize all images to a common reference kernel.

**Purpose:**

We explore training a harmonization model using paired reconstruction kernels (obtained from the same manufacturer for a given subject) and unpaired reconstruction kernels (obtained across different manufacturers for different subjects) in a low dose lung cancer screening cohort, validating our approach through quantitative CT measurements. Our overall goal is to use both sets of data to construct a shared latent space while recognizing the ability of different types of data to contribute through different classes of loss functions.

**Methods:**

We develop a multipath cycleGAN that enables multi‐domain kernel harmonization through a shared latent space, domain specific encoder‐decoder architectures, and discriminators trained in an unsupervised manner using a mixture of paired and unpaired data. We train our model using 100 scans each from seven representative kernels (Siemens B50f, Siemens B30f, GE BONE, GE STANDARD, GE LUNG, Philips C, and Philips D) from the National Lung Screening Trial (NLST) dataset, enabling harmonization across 42 different kernel combinations. Using 240 withheld scans from each kernel, we evaluate our approach on paired kernels using percent emphysema. For unpaired kernels, we harmonize all scans to the style of a reference soft kernel (Siemens B30f) and evaluate our model using percent emphysema, followed by a general linear model analysis that investigates the impact of age, sex, smoking status, and kernel on emphysema. Additionally, we harmonize all soft kernels to a reference hard kernel (Siemens B50f), quantifying percent emphysema. We assess anatomical consistency in unpaired kernels harmonized to a reference soft kernel by comparing segmentations of lung vessels, muscle, and subcutaneous adipose tissue derived from TotalSegmentator between the non‐harmonized and harmonized images. We compare the performance of our model to the standard cycleGAN and a switchable cycleGAN model.

**Results:**

For paired kernels, the proposed multipath approach reduced differences in percent emphysema as seen from the Bland Altman analysis (*p *< 0.05), achieving the best performance on one kernel pair over the standard cycleGAN and on two pairs over the switchable cycleGAN.For unpaired kernels where all source kernels were harmonized to a reference soft kernel, our method mitigated differences in three of six kernels, comparable to the switchable cycleGAN, while the standard cycleGAN mitigated differences in four of six kernels. When harmonizing all soft kernels to a reference hard kernel, our approach outperformed the switchable cycleGAN and was comparable to the standard cycleGAN. Our proposed approach maintains anatomical consistency in muscle, adipose tissue across all unpaired kernels and showed reasonable overlap on three kernels for lung vessels when compared to the cycleGAN; however, performance was lower when compared to the switchable cycleGAN.

**Conclusions:**

Paired and unpaired kernel harmonization with a shared latent space multipath cycleGAN mitigates errors in emphysema quantification and maintains anatomical consistency after harmonization.

## 1 INTRODUCTION

**FIGURE 1:**
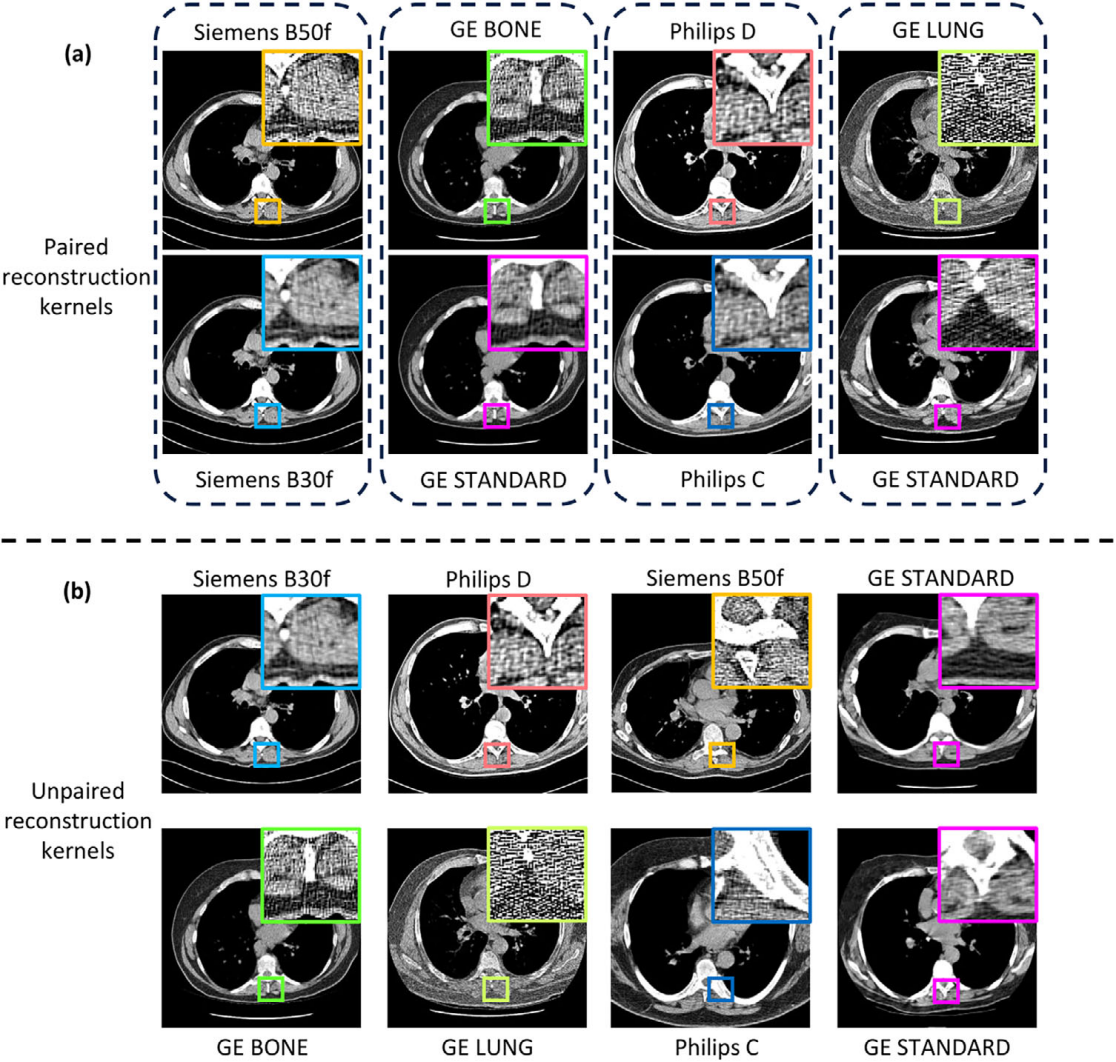
Reconstruction kernels influence the noise and resolution of the underlying anatomical structure in a computed tomography image. (a) Paired reconstruction kernels obtained from a given vendor exhibit a one‐to‐one pixel correspondence between the scans, which enables kernel harmonization. However, (b) across vendors, unpaired kernels show differences in anatomy, scan protocol, field of view, and reconstruction window. This creates additional difficulties that make harmonization a more challenging task.

In computed tomography (CT), there exists a trade‐off spectrum between spatial resolution and pixel noise: scans reconstructed with a “hard” kernel have higher spatial resolution and increased pixel noise while those reconstructed with a “soft” kernel have lower spatial resolution and decreased pixel noise^1^ (Figure 1). Hard kernels are beneficial when lung or bone is the anatomy of interest whereas soft kernels are beneficial for soft tissue like the mediastinum.^2^ However, the sharpness of the kernel impacts the strength of distinct spatial frequencies and image features; these changes result in inconsistent quantitative measurements across kernels.^3^ In thoracic imaging, emphysema quantification,^4^, ^5^ body composition assessment,^6^ coronary artery calcification^7^ and radiomic feature reproducibility^8^, ^9^ are sensitive to the choice of kernel. Similarly, in abdominal CT, kernel choice can influence the detection of hepatocellular carcinoma for different contrast phases.^10^ This variability in kernels is common in multi‐centre and longitudinal studies, making it difficult to compare scans. Therefore, it is important to harmonize images reconstructed with different kernels to an appropriate reference that suits the anatomy and task being investigated for reliable and consistent quantitative analysis.

Kernel harmonization is an approach that mitigates systematic differences in quantitative measurements due to the variability of reconstruction kernels. The goal of kernel harmonization is to standardize the pixel noise in images reconstructed with different kernels to a common reference kernel, ensuring comparable and consistent quantitative measures. Progress has been made to implement kernel harmonization through physics‐based and deep learning approaches. Physics based approaches generally involve the use of the point spread function (PSF) and modulation transfer function (MTF) that are modelled in the harmonizer. For instance, Ohkubo et al.^11^ performed kernel filtering between two kernels using the ratio of the MTFs derived from the PSF. The residual intensity obtained on the difference images for lung cancer screening images and phantoms were mitigated after harmonization. Similarly, Sotoudeh‐Paima et al.^12^ developed CT HARMONICA, a physics‐based harmonization method that harmonized CT images to isotropic resolutions of 0.75 × 0.75 × 0.75 mm and a global noise index described by Christianson et al.,^13^ to facilitate accurate emphysema quantification in phantoms and clinical data. By incorporating the MTF and an adaptive Wiener filter in their harmonizer, differences in emphysema‐based measurements were mitigated. Zarei et al.^14^, ^15^ developed a harmonizer that incorporated the MTF into a deep neural network for harmonizing phantom images. Their approach mitigated differences in emphysema measurements, improved the detectability index of tumours in clinical data and mitigated the standard deviation of noise that is, introduced by the kernel. While physics‐based approaches are beneficial for kernel harmonization, the MTF and PSF need to be empirically estimated from phantoms which may not be available in retrospective studies.

Deep learning‐based methods for kernel harmonization have shown potential for intra‐vendor and cross vendor harmonization using (a) supervised and (b) unsupervised learning. Supervised learning methods rely on “paired” reconstruction kernels, meaning images reconstructed with hard and soft kernels of the same subject having a one‐to‐one pixel correspondence. In contrast, unsupervised methods use “unpaired” reconstruction kernels obtained from different vendors where there exists a difference in the underlying anatomical alignment and acquisition protocol. Lee et al.^16^ developed a convolutional neural network (CNN) model inspired by Kim et al.^17^ that learned to predict a residual map between a source kernel and target kernel for different pairs of reconstruction kernels obtained from a Siemens manufacturer. The residual learning strategy effectively captured differences between kernels, enabling standardization of emphysema measurements without the need for projection data. Eun et al.^18^ leveraged super resolution networks and developed a squeeze excitation block network that learned to translate between reconstruction kernels obtained from a single vendor, showing improvements in image similarity metrics after harmonization. Inspired by the residual learning strategy, Choe et al.^19^ leveraged the CNN model for kernel harmonization and highlighted its importance in reproducing radiomic features for different kinds of pulmonary nodules. Jin et al.^20^ implemented a two‐stage approach involving a truncation restoration network that compensates for artefacts followed by a kernel harmonization network for paired kernels obtained from four different manufacturers, showing considerable improvements in emphysema quantification.

Within deep learning methods, image‐to‐image translation is another approach for kernel harmonization that aims to learn a mapping between a source image and a target image, changing the style of the image while maintaining the contents.^21^ Generative adversarial networks (such as pix2pix^22^ for paired data and cycleGAN^23^ for unpaired data) have gained popularity for kernel harmonization. One such approach was implemented by Selim et al.,^24^ where a novel dynamic window‐based training was implemented using a generative adversarial network. The trained model reproduced radiomic features and showed good performance on image similarity metrics. Tanabe et al.^25^ developed a pix2pix model that incorporated a region wise learning strategy on paired hard and soft kernels, minimizing differences in measurements for emphysema, body composition, and coronary calcium. Similarly, in our previous work,^26^ we incorporated the residual learning strategy into a pix2pix model and performed kernel harmonization on paired kernels obtained from a multi‐vendor setting for lung cancer screening. The proposed approach mitigated differences in emphysema and body composition measurements and further highlighted reproducibility of radiomic features after harmonization. While CNNs and pix2pix models are reliable for kernel harmonization, they are limited to paired data from a given vendor, requiring multiple models for different pairs.

Unpaired kernel harmonization is an active area of research, posing challenges that include differences in image acquisition protocols and differences in the anatomical alignment of subjects across different manufacturers. Recent progress has facilitated cross vendor harmonization across unpaired kernels. For instance, Yang et al.^27^ demonstrated kernel harmonization using a novel switchable cycleGAN model for head and facial bone kernels. Similarly, Selim et al.^28^ developed a cross‐vendor harmonization model using a cycleGAN that involved a self‐attention module in the generator followed by a feature‐based domain loss that helped in downstream radiomic feature assessment. Their approach improved the reproducibility of radiomic features with improved image similarity metrics. Gravina et al.^29^ leveraged a cycleGAN that performed kernel harmonization on two kernels obtained for PET data, with their approach improving performance on image‐based similarity metrics.

Existing approaches investigate paired and unpaired CT kernel harmonization by learning representational spaces that pertain to specific pairs. We propose that kernels obtained from various vendors can be jointly represented in a single latent space. Herein, we investigate paired and unpaired CT kernel harmonization across different combinations of reconstruction kernels in a low‐dose lung cancer screening dataset with images obtained from multiple vendors using a proposed multipath cycleGAN trained in two stages. The key idea behind the multipath cycleGAN lies in the use of domain specific encoder‐decoder architectures, stitched together through a shared latent space. We hypothesize that a shared latent space will:

1. Enforce better consistency in emphysema measurements for paired reconstruction kernels as compared to separate latent spaces such as traditional cycleGAN^23^.
2. Mitigate kernel effects in emphysema quantification when all kernels are harmonized to a reference soft kernel, achieving better consistency compared to separate latent spaces for every domain.
3. Mitigate kernel effects in emphysema quantification when all soft kernels are harmonized to a reference hard kernel.
4. Enforce anatomical consistency for segmentations on the harmonized images for all paths that involve unpaired kernel harmonization to a reference soft kernel.

We compare our model with existing approaches that perform kernel harmonization which include the traditional cycleGAN^23^ model that uses two separate latent spaces for image‐to‐image translation and the switchable cycleGAN model implemented by Yang et al.^27^ that involves a cycleGAN model with a single generator. These baseline models are trained for all the paired reconstruction kernels, all paths that involve harmonization to a reference soft kernel, and all soft kernels harmonized to a reference hard kernel. The performance of the baselines are compared with our multipath cycleGAN model for the following tasks: (a) emphysema quantification on paired kernels (b) emphysema quantification of all kernels harmonized to a reference soft kernel (c) Impact of kernel on emphysema measurements using a general linear model, and (d) anatomical consistency on the harmonized images for the unpaired kernels using TotalSegmentator.^30^

## 2 METHODS

Inspired by prior research on paired and unpaired kernel harmonization, we leverage the cycleGAN model to develop a multipath cycleGAN model that harmonizes paired and unpaired kernels in a low dose lung cancer screening population from multiple vendors through a shared latent space trained in two stages. In our previous work,^31^ we developed a multipath cycleGAN model to address paired and unpaired CT kernel harmonization for images reconstructed from Siemens and GE scanners. This approach introduced a shared latent space that allowed for harmonization across different combinations of reconstruction kernels using domain specific encoder‐decoder architectures and discriminators.

We expand upon our previous work by proposing a two‐stage multipath cycleGAN model that involves a larger dataset from various kernels and standardized field of view across all images. In Stage one, we harmonize across all possible combinations of reconstruction kernels obtained from the Siemens and GE vendors. We incorporate an additional loss function between the input source kernel and output target kernel for a given path to ensure that the radio‐opacity of the images do not shift during harmonization. In Stage two, we freeze the encoders and decoders trained in Stage one and train all discriminators and the networks for the Stage two kernels to facilitate harmonization across all combinations of kernels from the Philips and GE vendors. We develop a strategy to select the best performance of the model on the validation data using emphysema scores and evaluate the model's ability to mitigate differences in emphysema scores for the paired and unpaired kernels. Additionally, we study the impact of age, sex, current smoking status, and kernel on all the kernels that were harmonized to a reference soft kernel using a general linear model. We quantify anatomical consistency between the input source kernel and harmonized images across all the unpaired reconstruction kernel paths using Dice scores obtained on skeletal muscle, lung vessels, and subcutaneous adipose tissue.

### 2.1 Data selection

We harmonize CT images reconstructed using kernels from the National Lung Screening Trial (NLST), a randomized control trial that screened patients for lung cancer using low‐dose CT (LDCT) scans and chest radiography scans.^32^ Participants involved in this study were in the age range of 55–74 years, had a smoking history of 30 pack years or more and had last smoked within 15 years.^32^ Each participant underwent three screenings, labelled T0, T1 and T2 at one‐year intervals, with T0 considered as the baseline. We consider LDCT scans from the baseline that were reconstructed with different types of reconstruction kernels from multiple vendors. In a specific vendor, we choose subjects having a scan reconstructed using both a “hard” kernel and a “soft” kernel, following the NLST protocol,^33^ also known as “paired” reconstructions, where a one‐to‐one pixel correspondence is present between the scans. Across two vendors, there exists no overlap between subjects, resulting in “unpaired” reconstructions that lack a one‐to‐one pixel correspondence (Figure 1). We identify the following representative reconstruction kernel pairs: B50f (hard) and B30f (soft) from the Siemens vendor, D (hard) and C (soft) from the Philips vendor, BONE (hard), LUNG (hard), and STANDARD (soft) from the GE vendor. The images obtained from all vendors in the NLST were reconstructed with vendor‐specific algorithms for filtered back projection.^34^, ^35^ Subjects having scans reconstructed with the STANDARD kernel are either paired with the BONE kernel or the LUNG kernel. All images were acquired with a peak kilovoltage of 120–140 kVp, tube current of 40‐80 mAs, detector collimation ≤ 2.5 mm, pitch of 1.0–2.5, slice thickness of 1.0–3.2 mm, and reconstruction interval of 1.0–2.5 mm.^34^, ^36^ Although subjects have scans reconstructed using the same projection data, the spatial alignment captured in the field‐of‐view (FOV) might be different. Therefore, while selecting paired reconstruction kernels, we conducted a manual quality assurance to include scans that had the same spatial alignment.^26^ For every reconstruction kernel, we choose 100 volumetric scans that were sampled at random without replacement to train our model. A detailed list of the reconstruction kernels and the number of images used for training have been provided in Table 1. We select 100 volumes from each kernel for validation to select the optimal checkpoint for generalization. We perform inference on 240 volumes from the withheld test dataset for every reconstruction kernel based on performance on the validation dataset.

**TABLE 1:** Reconstruction kernels from four different manufacturers are used for kernel harmonization. For a given manufacturer, there exists paired reconstruction kernels that have a one‐to‐one pixel correspondence. However, subjects across manufacturers remained unpaired, resulting in difference in protocol and anatomy. The available data is used to train paired as well unpaired reconstruction kernels in an unpaired manner.

Manufacturer	Reconstruction kernel	No. of volumes for training	Total number of training slices
Siemens	B50f (hard)	100	16343
	B30f (soft)	100	16343
GE	BONE (hard)	100	14614
	STANDARD (soft)	100	14614
	LUNG (hard)	100	15723
	STANDARD (soft)	100	15723
Philips	D (hard)	100	19359
	C (soft)	100	19359

### 2.2 Preprocessing

We train our model on axial slices of size 512 × 512 pixels. In our previous work,^31^ we observed that the display field‐of‐view (DFOV) for the Siemens and GE scans were different, resulting in artifacts that were generated outside the DFOV in the harmonized GE scans. Therefore, we generate a binary mask by inscribing a circle of radius 256 pixels centered in the image where all pixels within this circle are set to one and the remaining are set to zero. We apply this binary mask to all the Siemens and Philips scans, standardizing the DFOV to be circular for all kernels. We clip the images in the range of [−1024, 3072] Hounsfield units (HU) and normalize them to the range of [−1,1].^26^

### 2.3 Model architecture: Multipath cycle GAN

**FIGURE 2:**
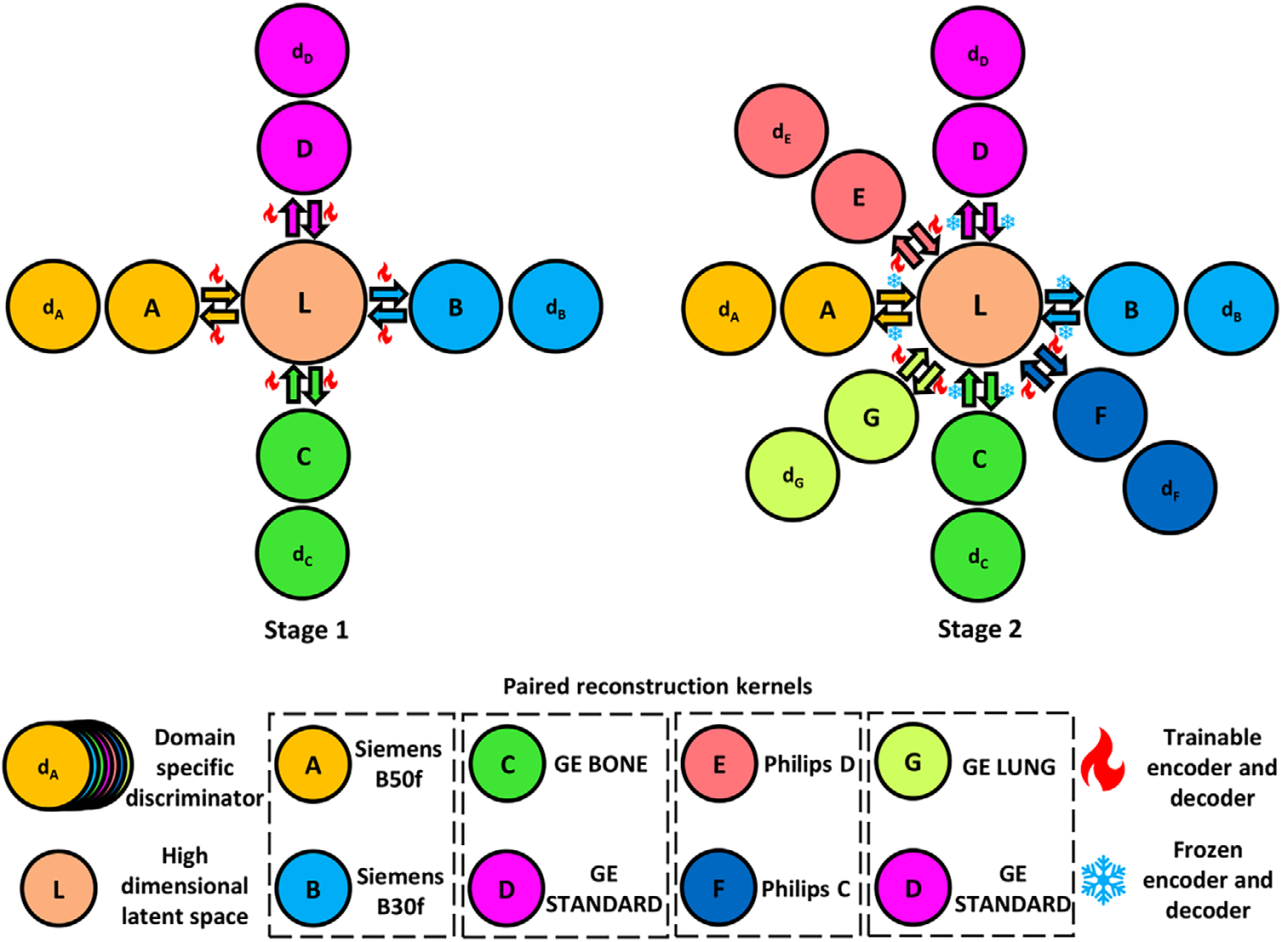
We hypothesize that inter‐vendor and intra‐vendor harmonization across different combinations of reconstruction kernels can be performed using a multipath cycleGAN model, trained in two stages. The paired reconstruction kernels are grouped together based on the manufacturer, while all other combinations denote unpaired kernels. In Stage one, we harmonize across all possible combinations of four different reconstruction kernels through a high dimensional shared latent space (denoted by L) using domain specific encoder‐decoder architectures. In Stage two, we freeze the trained encoders and decoders and train the model to account for new combinations across all available reconstruction kernels.

In a cycleGAN model, the forward and backward path use an individual latent space for image‐to‐image translation using two generators and two discriminators. However, when the number of domains is greater than two, multiple cycleGAN models need to be implemented for every combination of source and reference domains. We model a shared latent space that allows for multi‐domain kernel harmonization using a multipath cycleGAN comprising domain specific encoder‐decoder pairs that form generators and domain specific discriminators that take part in the adversarial training. Figure 2 illustrates the proposed multipath cycleGAN that builds upon the traditional cycleGAN^23^ model. We train this model across all possible combinations of reconstruction kernels in two separate stages. In Stage one, we facilitate harmonization across paired and unpaired kernels in an unsupervised manner between different combinations of reconstruction kernels obtained from the Siemens and GE manufacturers where all the encoders, decoders and discriminators are jointly trained. In Stage two, we introduce kernels from the GE and Philips manufacturers, instantiating encoders, decoders, and discriminators that are trainable. We freeze the encoders and decoders of the domains trained in Stage one while the discriminators are trainable to ensure that new kernels are harmonized to the style of all Stage one kernels. Freezing the encoders/decoders prevents catastrophic forgetting of the previously learned mappings, ensuring that we can adapt the new kernels to the style of the old kernels without degrading the performance on previously harmonized domains. Therefore, Stage one establishes a shared latent space that can be leveraged by the models in Stage two to allow for harmonization of all new kernels to the style of the old kernels. All paths between the old and new kernels use supervised domain adaptation to align the Stage two kernels to the style of the Stage one kernels. Here, supervised domain adaptation refers to anchoring the new domains to the fixed Stage one models. All the paths between the new kernels are trained in an unsupervised manner.

We build each domain specific generator by breaking down a ResNet model into its corresponding source encoder and target decoder pair that performs image translation through a shared latent space. The ResNet model is a fully convolutional model, adopted from the traditional cycleGAN model, which itself is based on the architecture proposed by Johnson et al.^37^ The ResNet model uses strided and fractionally strided convolutions to upsample and downsample the image. The first layer of the encoder uses a 7 × 7 Convolution‐InstanceNorm‐ReLU layer with a stride of one followed by two 3 × 3 Convolution‐InstanceNorm‐ReLU layers with a stride of two. The shared latent space is composed of nine residual blocks where each residual block is a convolution block with skip connections comprising of two 3 × 3 convolution layers, instance normalization and ReLU layers. The decoder uses two 3 × 3 fractional strided Convolution‐InstanceNorm‐ReLU layers followed by a 7 × 7 fractional strided Convolution‐InstanceNorm‐ReLU layer. We include additional details of the ResNet model in Figure S1. Every domain specific source encoder maps the input of size N × 1 × 512 × 512 to a shared latent space, resulting in a feature vector of size N × 256 × 128 × 128 where N denotes the batch size. The domain specific target decoder translates the latent feature vector to an image in the style of the target domain with the same size as the input. The discriminator is a PatchGAN^22^ that classifies 70 × 70 patches of images as real or synthetic. The implemented discriminator model is a fully convolutional network consisting of three blocks where each block consists of 4 × 4 Convolution‐InstanceNorm‐LeakyRELU layers with a stride of two. A convolution is applied after the last layer to obtain a single channel prediction map. The 70 × 70 patches are generated because of the implicit receptive field that arises from the convolution operation which relies on the kernel size and stride. The setup described facilitates encoder‐decoder architectures that stitch all possible reconstruction kernel pairs for multi domain image‐to‐image translation.

**FIGURE 3:**
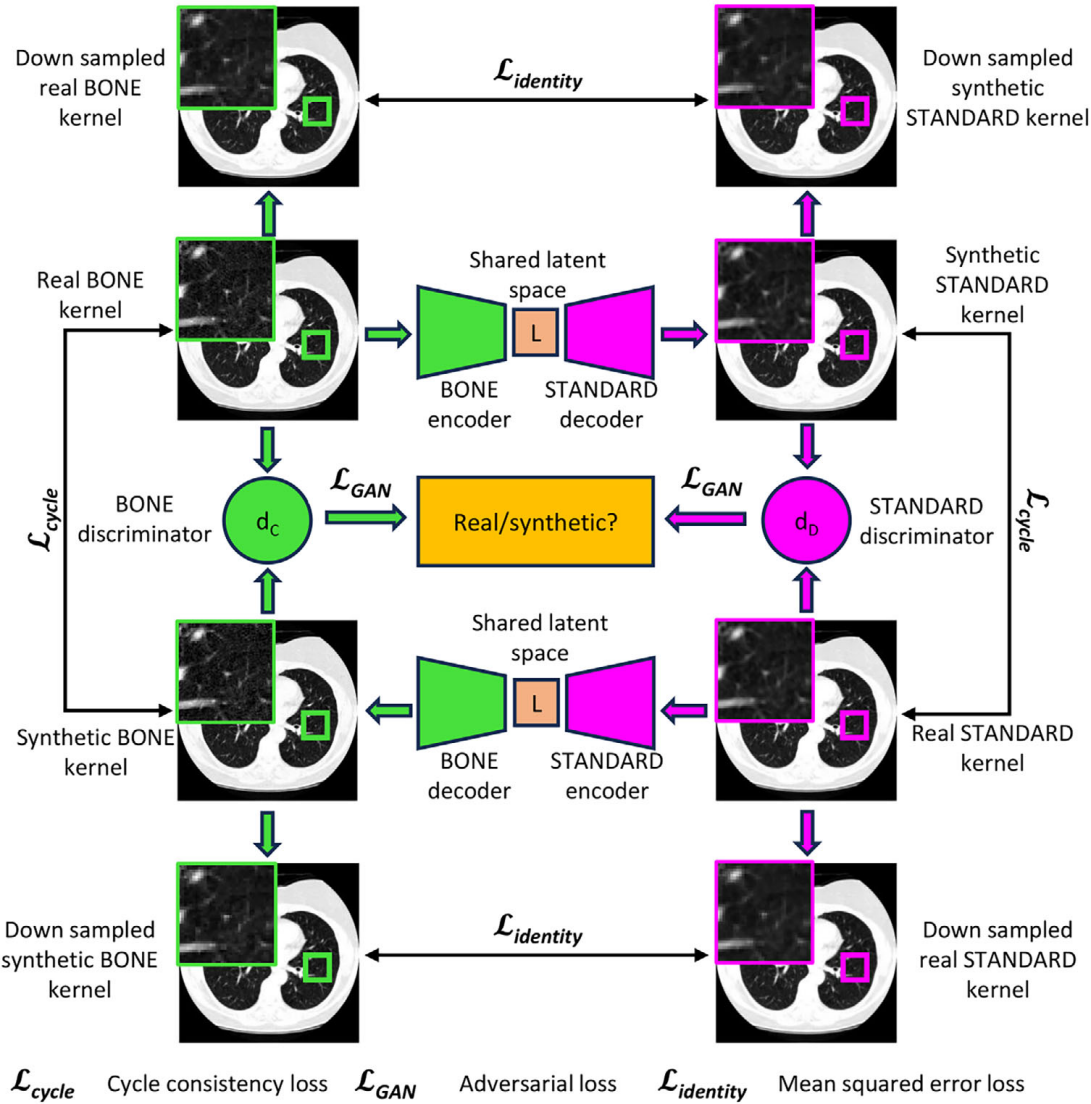
For any given pair of reconstruction kernels, there exists a forward and backward path. The generator is a ResNet, formed by a source encoder and target decoder in the forward path and a target encoder and source decoder in the backward path. Each generator produces a synthetic image with the style of either the source or target kernel. A PatchGAN is used as a discriminator for the corresponding domain to distinguish between real and synthetic images. In addition to the adversarial and cycle consistency losses, an identity L2 loss is applied between the down sampled real and synthetic image to prevent a shift in the radio‐opacity of the images.

For any given combination of reconstruction kernels, we construct a cycleGAN using the corresponding source and target encoders, decoders, and discriminators. We illustrate one such path that involves images reconstructed with the BONE and STANDARD kernels in Figure 3. In the forward path, the BONE encoder maps the real BONE image into the shared latent space, and the STANDARD decoder produces a synthetic STANDARD image while in the backward path, the STANDARD encoder maps the real STANDARD image through the shared latent space and the BONE decoder produces a synthetic BONE image. The BONE and STANDARD discriminators evaluate whether the images produced in the cyclic path are real or synthetic. We introduce an “identity” loss function, denoted by $L_{\mathrm{identity}}$ to the cycleGAN objective to prevent a shift in the radio‐opacity of the synthetic image. This loss function is computed as the mean squared error (MSE) between the down sampled versions of the real and synthetic images in the forward and backward paths. Down sampling the images smoothens the kernel, thereby allowing the loss to guide the model in preserving the radio‐opacity.

### 2.4 Two stage training approach

In Stage one, we train our models using images reconstructed using the Siemens B50f, Siemens B30f, GE BONE and GE STANDARD kernels. The encoder‐decoder pairs are trained across 12 different paths for 6 possible pairs of reconstruction kernels. We train this model in parallel on two NVIDIA RTX A6000 GPUs for 120 epochs using a batch size of two and an Adam^38^ optimizer for the generator and discriminator. The learning rate was 0.002 for the first 60 epochs and decayed linearly to zero for the remaining 60 epochs. We continue training our model from epoch 120 to epoch 200 with a fixed learning rate of 0.002 to obtain additional checkpoints for evaluation. The generator and discriminator are governed by an adversarial loss, implemented using the LSGAN^39^ loss function. A cycle consistency loss is implemented to ensure domain consistency during image translation. We weight the cycle consistency loss $\lambda_{\mathrm{cycle}}$ to 10 for the forward and backward path, following the default cycleGAN. A squared L2 norm identity loss is computed between the down sampled versions of the real and synthetic images. The real and synthetic images are down sampled to 256 × 256 pixels using bilinear interpolation. This identity loss is weighted by a factor $\lambda_{\mathrm{identity}}$. In the first epoch, $\lambda_{\mathrm{identity}}$ is set to 10^6^ such that the generators behave as identity networks, producing a reconstructed version of the input. In the subsequent epochs, we scale down the weighting factor by 100 and fix $\lambda_{\mathrm{identity}}$ to 0.01 at the sixth epoch such that the adversarial loss takes over the training. We implement a total of 12 adversarial losses, 12 cycle losses, and 12 identity losses in Stage one.

In Stage two, we introduce images reconstructed using the GE LUNG, Philips D, and Philips C kernels. The GE STANDARD kernels paired with the GE LUNG kernels are used in Stage two of model training. We freeze the encoders and decoders for the Siemens B50f, Siemens B30f, GE BONE, and GE STANDARD kernels. The encoders and decoders from Stage one are frozen to promote domain adaptation of the new kernels to the pre trained kernels. We load the trained weights for the encoders, decoders, and discriminators trained in Stage one based on the performance of the best epoch on the validation dataset. The best epoch is defined as the training epoch that achieves optimal performance in mitigating differences in emphysema quantification across paired and unpaired kernels using a combination of mean squared error (MSE) and Kullback–Leibler divergence. We compute the best epoch on the 100 volumes for each kernel from the validation dataset. The new models introduced in Stage two are initialized with the average of the pre‐trained weights using the respective encoders, decoders and discriminators. The encoder‐decoder pairs are trained across 30 different paths for 15 possible pairs of reconstruction kernels. With the presence of many images for every domain, training across all datasets was prohibitively expensive. Therefore, we randomly sampled 20% of the images from every available dataset in every epoch and trained using the same hardware and optimization settings as Stage one. The learning rate was 0.002 for the first 100 epochs and decayed linearly to zero for the remaining 100 epochs. We implemented 30 adversarial losses, 30 cycle losses, and 30 identity losses in Stage two.

### 2.5 Baseline models

We implement the traditional cycleGAN^23^ model and the switchable cycleGAN model^27^ as baselines for comparison against our model. We implement four individual cycleGAN models for the paired kernels and five individual cycleGAN models for the unpaired kernels that correspond to the path that harmonizes to a reference Siemens B30f kernel for every baseline. For the paths that involve harmonization of soft kernels to a reference hard kernel, we train three individual cycleGAN models and switchable cycleGAN models. We trained each traditional cycleGAN on a single NVIDIA RTX A6000 GPU for 120 epochs using a batch size of six, Adam optimizer for the generator and discriminator, and learning rate of 0.002. We use a batch size of six as this was the largest number of samples we could accommodate while training the traditional cycleGAN. We use a linear learning rate scheduler to tune the optimizer, where the learning rate remains constant for the first 60 epochs and decays linearly till it reaches zero for the remaining 60 epochs. With the availability of minimal checkpoints for the baselines, we continue training the cycleGAN model from epoch 120 to epoch 200 with a fixed learning rate of 0.002 to obtain additional checkpoints for model evaluation. We trained every switchable cycleGAN model on an NVIDIA Quadro RTX 5000 GPU for 200 epochs using the default model configurations, which involved a batch size of 16, patch size of 128 × 128 pixels, Adam optimizer for the generator and discriminator, and the learning rate of 1 × 10^−5^. The models trained for soft to hard kernel harmonization followed the same configuration.

## 3 PERFORMANCE AND STATISTICS

### 3.1 Emphysema quantification

We assess the efficacy of harmonization for the baselines and multipath cycleGAN model using percent emphysema quantification. We automatically identify regions in the lungs and obtain lung masks using a publicly available algorithm.^40^ The density of areas affected with emphysema on CT images ranges from −900 to −1024 HU.^41^ We quantify the percentage of voxels in the lung region that have a radio‐opacity less than –950 HU using the available lung masks and obtain percent emphysema scores. This approach of thresholding to compute percent emphysema is also known as low attenuation area (LAA). A threshold of –950 HU is considered optimal since it reflects the macroscopic pathological features of emphysema.^42^, ^43^ For the paired kernels obtained from a given manufacturer, we harmonize to the corresponding soft kernel, while for unpaired kernels, we harmonize from a source kernel to a reference Siemens B30f kernel and measure percent emphysema. Similarly, we harmonize all the soft kernels to a reference Siemens B50f kernel and evaluate percent emphysema.

### 3.2 Epoch selection for model selection

To choose an epoch for model generalizability, we use emphysema quantification as a validation metric for all models. In Stage one, we consider Siemens B50f to Siemens B30f (paired), GE BONE to Siemens B30f (unpaired), and GE STANDARD to Siemens B30f (unpaired), for the purpose of epoch selection. We compute MSE between the emphysema scores obtained on the paired kernels and KL divergence between the emphysema scores obtained on the unpaired kernels. When performing model selection on validation data, we considered both point‐to‐point matching and distribution matching as our criteria, placing greater emphasis on measurement with paired data with larger coefficients. This design choice ensures that the selected model balances the ability to mitigate differences in emphysema measurements for both paired and unpaired kernels. We rank the MSE and KL divergence scores using the *rankdata* function from the *scipy* python package and perform a weighted sum of ranks using the formula:

(1)

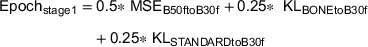




In Stage two, we consider Philips C to Siemens B30f (unpaired), Philips D to Siemens B30f (unpaired), GE LUNG to B30f (unpaired), GE LUNG to GE STANDARD (paired) and Philips D to Philips C (paired). Since there are two paired domains and three unpaired domains, the coefficients for MSE and KL are balanced to ensure equal contribution to weight selection. The choice of equal weights is equivalent to summing all ranks and dividing by a factor of five. This balanced ratio is an explicit design choice. We rank the MSE and KL divergences using the formula:

(2) $$\begin{array}{cc} \mathrm{Epoc}h_{\mathrm{stage}2} & =0.2*\mathrm{MS}E_{\mathrm{LUNGtoSTANDARD}}+0.2*\mathrm{MS}E_{\mathrm{DtoC}}\\ & \;+0.2*\;KL_{\mathrm{DtoB}30f}+0.2*KL_{\mathrm{CtoB}30f}\\ & \;+0.2*KL_{\mathrm{LUNGtoB}30f} \end{array}$$

In the case of soft to hard kernel harmonization, for Stage one, we choose Siemens B30f to B50f (paired) and STANDARD to B50f (unpaired) while for Stage two we choose Philips C to Siemens B50f (unpaired). We use a similar design criterion in Stage one to weight the terms, ensuring balanced epoch selection. In Stage two, we use only one term to select our epoch. We pick the best epoch using the following equations:

(3) $$\begin{array}{cc} \mathrm{Epoc}h_{\mathrm{stage}1} & =0.5*\mathrm{MS}E_{B30\mathrm{ftoB}50f}\\ & \;+0.5*KL_{\mathrm{STANDARDtoB}30f} \end{array}$$

(4) $$\mathrm{Epoc}h_{\mathrm{stage}2}=KL_{\mathrm{CtoB}50f}$$

Using the above equations, we obtain the overall combined ranks and select the epoch with the lowest rank as the best epoch for model inference. When harmonizing all kernels to a reference soft kernel, we select the following epochs across all models for Stage one and two: 100th and 119th epoch for the multipath cycleGAN, 120th epoch and 118th epoch for the standard cycleGAN and 190th epoch and 90th epoch for the switchable cycleGAN. In the case of harmonization from soft to hard kernels, we selected the 80th and 100th epoch from Stage one and two for the multipath cycleGAN. For the standard cycleGAN model, we selected the 162nd and 118th epoch, and the 70th and 145th epoch for the switchable cycleGAN model.

### 3.3 Statistical methods

We quantify emphysema measurements using the method described in Section 3.1 and obtain percent emphysema scores on the hard kernel image, soft kernel image and the harmonized image. We observe the agreement between percent emphysema measurements before and after harmonization on all the paired reconstruction kernel images using Bland‐Altman^44^ style plots. We compute the median root mean square error (RMSE) and 95% confidence intervals (CI) using bootstrapping.^45^ We use 1000 resamples where a random sample is obtained with replacement to form the bootstrap distribution. For every resample, the test statistic was computed, and the confidence intervals of the bootstrap distribution was obtained. For the unpaired reconstruction kernels, we assess statistical significance between emphysema measurements before and after harmonization using the Mann–Whitney U test.^46^ After harmonizing all source kernels to a reference target soft kernel, we investigate the impact of age, sex, current smoking status and kernel on the emphysema measurement using a general linear model given by the equation:

(5) $$\begin{array}{c} Y\;∼\;\beta_{0}+\beta_{1}*X_{1}+\beta_{2}*\;X_{2}+\beta_{3}*\;X_{3}+\beta_{4}*X_{4}+\varepsilon \end{array}$$

where Y is the percent emphysema measurement, $\beta_{0}$ is the intercept, $X_{1},X_{2},X_{3},X_{4}$ represent age, sex, reconstruction kernel and current smoking status of the subjects, $\beta_{1}$, $\beta_{2}$, $\beta_{3}$, $\beta_{4}$ are regression coefficients and ε denotes error.

### 3.4 Assessment of anatomical consistency before and after harmonization using TotalSegmentator

When training a cycle GAN model to translate an image from one domain to another, the use of distribution matching losses can lead to hallucinations where the model can introduce or remove features.^47^ Therefore, it is necessary to quantify hallucinations that occur during harmonization of unpaired reconstruction kernels due to anatomical variations across subjects scanned on two different vendors. We assess consistency of the lung vessels, skeletal muscle and subcutaneous adipose tissue (SAT) before and after harmonization using TotalSegmentator.^30^ Specifically, we use the tissue‐types model of TotalSegmentator and the lung vessels model,^48^ generating segmentations of lung vessels, muscle and SAT for the baselines and multipath cycleGAN across all the paths that harmonize a source kernel to a reference Siemens B30f kernel. We compute the Dice scores for lung vessels, muscle and SAT between the segmentations obtained from the same image before and after harmonization. We quantify the effect of the hallucinations between the baselines and multipath cycleGAN model by computing the Cohen's d statistic^49^ between the respective lung muscles, skeletal muscle and SAT Dice scores. The effect size is small when Cohen's d = 0.2, medium when Cohen's d = 0.5 and large when Cohen's d = 0.8. A positive Cohen's d indicated that group one had a higher mean than group two while a negative Cohen's d indicated the opposite.

## 4 RESULTS

### 4.1 Harmonization of paired reconstruction kernels

**FIGURE 4:**
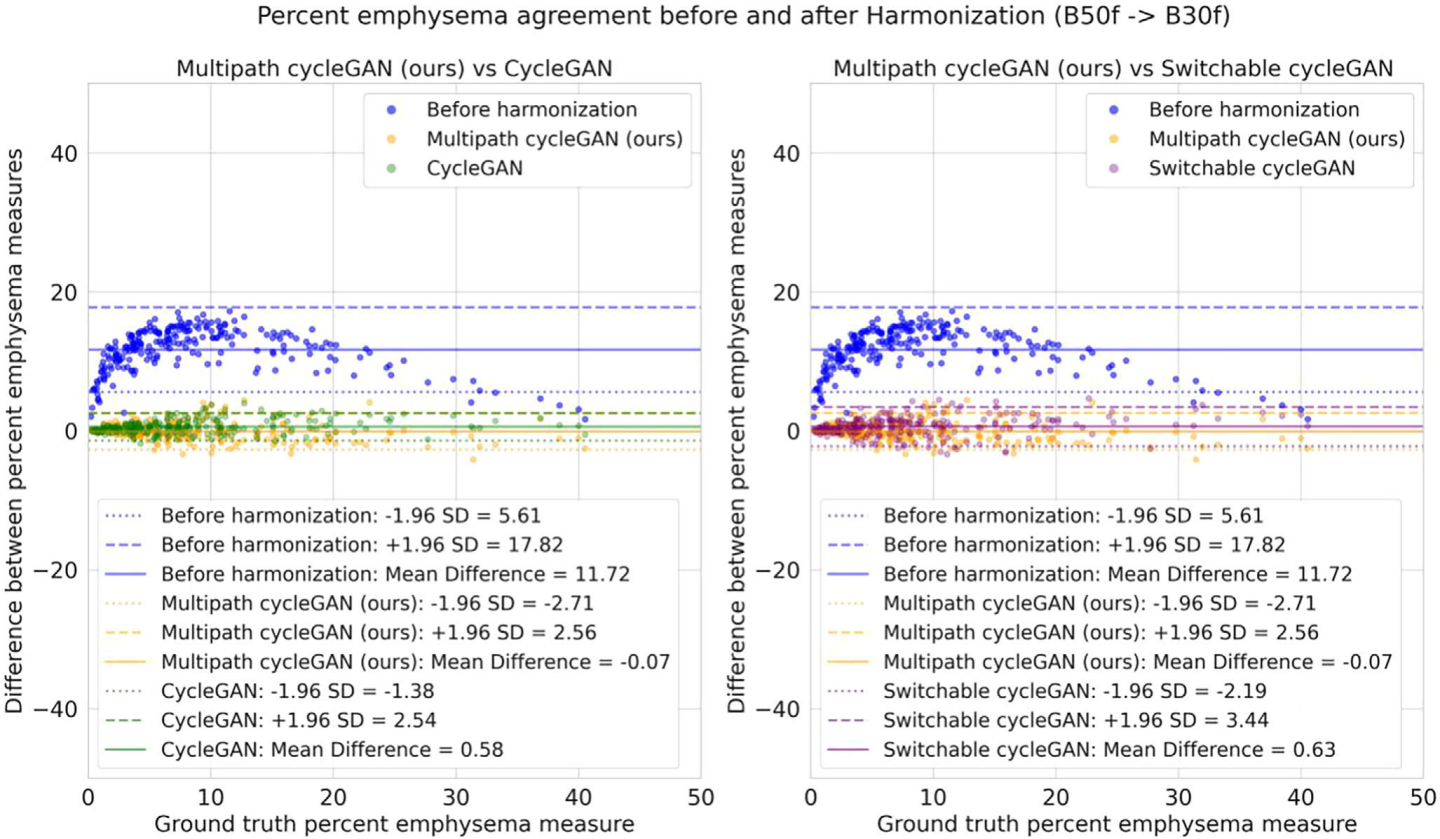
Bland Altman style plots are used to represent the agreement on emphysema measurements between paired hard and soft reconstruction kernels before and after harmonization. The solid line represents the mean difference between the emphysema measurements, and the dashed lines represent the standard deviation (SD) as 95% confidence intervals [−1.96SD, +1.96SD]. Blue represents measurements without harmonization, yellow represents the multipath cycleGAN, green represents the standard cycleGAN, and purple represents the switchable cycleGAN. Emphysema measurements disagree before harmonization. After harmonization, all models show improvements in agreements. The mean difference is close to zero, with the multipath cycleGAN showing better agreement on the Siemens kernels.

We present all median RMSE values and 95% confidence intervals for the emphysema scores obtained on the paired data in Table 2. We summarize the overall performance by reporting the proportional decrease in median RMSE after harmonization, expressed as a percentage using the median RMSE values in Table 2. Across all pairs, harmonization reduced the median RMSE substantially. The largest improvement in RMSE was seen in the (LUNG, STANDARD) pair across all models with the cycleGAN achieving the largest reduction in RMSE of 96.71%. On the (BONE, STANDARD) pair, the multipath cycleGAN obtained the largest reduction in RMSE (91.52%) while the cycleGAN achieved the largest reduction in RMSE on the (B50f, B30f) and (C, D) pairs. We visualize the RMSE measurements using a Bland Altman style plot for the (B50f, B30f) pair in Figure 4 with the corresponding plots for the remaining pairs in the supplementary material. Wilcoxon signed rank tests conducted between the measurements before and after measurements were statistically significant (*p *< 0.05). While the proposed method mitigated differences in paired kernels, the standard cycleGAN achieved better performance on the (B50f, B30f), (D, C) and (LUNG, STANDARD) pairs. The switchable cycleGAN achieved better scores on the (D, C) and the (LUNG, STANDARD) pairs.

**TABLE 2:** Comparison of difference in percent emphysema measurements between paired reconstruction kernels before and after harmonization using standard cycleGAN, switchable cycleGAN, and multipath cycleGAN (ours).

Pairs	Difference in percent emphysema			
	Before harmonization	cycleGAN harmonization	Switchable cycleGAN harmonization	Multipath cycleGAN harmonization (ours)
(B50f, B30f)	12.12 (11.76, 12.42)	1.16 (1.05, 1.29)	1.57 (1.41, 1.71)	1.35 (1.22, 1.52)
(BONE, STANDARD)	9.91 (9.50, 10.33)	0.91 (0.81, 1.05)	1.01 (0.89, 1.21)	0.84 (0.75, 0.95)
(D, C)	15.81 (15.28, 16.33)	1.31 (1.18, 1.49)	1.78 (1.57, 2.12)	2.47 (2.11, 2.93)
(LUNG, STANDARD)	19.20 (18.58, 19.78)	0.63 (0.52, 0.75)	0.99 (0.78, 1.33)	1.05 (0.83, 1.46)

*Note*: We present the root mean squared error (RMSE) computed between the source kernel and the target kernel pair followed by 95% confidence intervals. Kernel harmonization significantly reduces difference in measurements before and after harmonization (*p* < 0.05, Wilcoxon signed‐rank test with bootstrapping). Our approach achieves comparable performance with baselines for most of the paired kernels, demonstrating the effectiveness of a shared latent space.

### 4.2 Harmonization of all unpaired reconstruction kernels to a reference soft kernel

**FIGURE 5:**
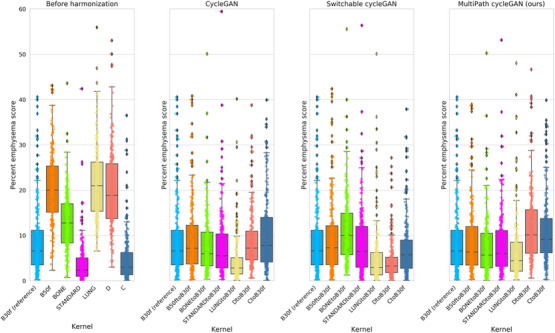
Unpaired CT kernels are challenging to harmonize due to the difference in scanner protocol, anatomy, and field of view. Before harmonization, hard kernels have a higher range of emphysema scores compared to soft kernels, that are preferred for emphysema. The reference Siemens B30f kernel is represented by the light blue box and strip plot. Harmonization of all kernels to the reference kernel mitigates differences in emphysema measurement, as evidenced by closer medians to the reference. The cycleGAN models show the best performance in mitigating differences in measurements across kernels, followed by the multipath cycleGAN and switchable cycleGAN models.

We harmonize all the source kernels to the reference Siemens B30f soft kernel. While the Siemens B50f kernel has paired data with the reference, all other source kernels are unpaired. We visualize the emphysema distributions before and after harmonization using boxplots overlaid with strip plots as seen in Figure 5. We represent all measurements as the median emphysema measurements. The reference Siemens B30f kernel, had a median of 6.60%. Before harmonization, Siemens B50f, GE BONE, GE LUNG and Philips D overestimated emphysema measurements with median scores of 20.02%, 12.75%, 21.01% and 18.87%. In contrast, GE STANDARD and Philips C had measurements of 2.34% and 2.96%, underestimating emphysema compared to the reference.

After harmonization, all models reduced variability in emphysema measurements and brought most distributions closer to the reference. The cycleGAN aligned the emphysema distributions for Siemens B50f, GE BONE, GE STANDARD, Philips D and Philips C close to the reference median but was less effective for GE LUNG (median 2.84%). The switchable cycleGAN showed improvements for Siemens B50f, GE STANDARD and Philips C but was less effective on GE BONE (median 10.01%), GE LUNG (median 3.28%) and Philips D (median 5.74%). The multipath cycleGAN model showed improvements across Siemens B50f, GE BONE, GE STANDARD and GE LUNG but overcorrected Philips D (median 10.13%) and Philips C (9.13%). Our proposed approach achieves comparable performance on Stage one kernels but shows variability in performance on Stage two kernels when compared to the baseline models.

### 4.3 Harmonization of all soft kernels to a reference hard kernel

We harmonize all soft kernels to the reference Siemens B50f hard kernel where the Siemens B30f kernel was paired with the reference. In Section 4.2, we considered the Siemens B30f as our reference kernel. For this task, the Siemens B30f is a source kernel that will be harmonized to the reference Siemens B50f kernel. The median emphysema scores for the reference B50f kernel was 20.02%. Before harmonization, the median scores for Siemens B30f, Philips C and GE STANDARD were 6.60%, 2.96% and 2.34% respectively (Figure S8). After harmonization, the distribution of all the soft kernels was closer to the reference hard kernel. The cycleGAN and multipath cycleGAN showed similar trends in performance on all the kernels with minimal differences in the median emphysema score. However, the switchable cycleGAN model underperformed by a small margin on the Siemens B30f and GE STANDARD kernels but achieved a median closer to the reference for the Philips C kernel (median 18.10%) when compared to the other models.

### 4.4 Qualitative results

**FIGURE 6:**
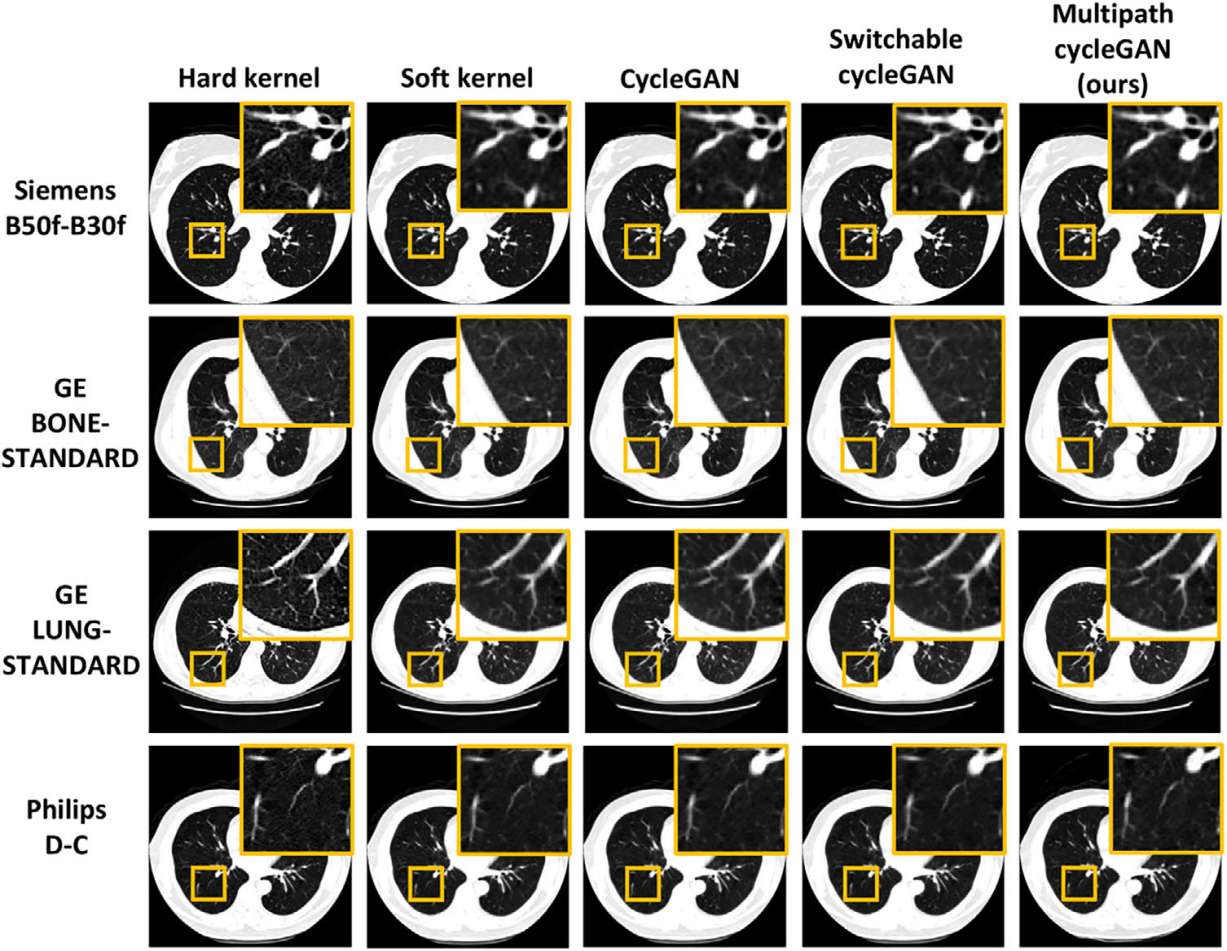
Paired kernels exhibit a one‐to‐one pixel correspondence between the hard and soft kernel in each vendor with differences in the pixel noise. Hard kernels sharpen the image while soft kernels smoothen the image. Harmonizing to the corresponding soft kernel enforces qualitative consistency in the lung parenchyma for the multipath cycleGAN that is comparable to the baseline models.

In the case of paired reconstruction kernels, we consider a specific region of the lung parenchyma for a subject having scans reconstructed with a hard and soft kernel within a given vendor. Harmonization from a hard kernel to a soft kernel within a given vendor enforces consistency in the texture of the selected lung region. The baseline approach requires models bespoke to each pair. In contrast, our multipath cycleGAN learns a shared latent space enabling harmonization across all paired kernels as seen in Figure 6.

**FIGURE 7:**
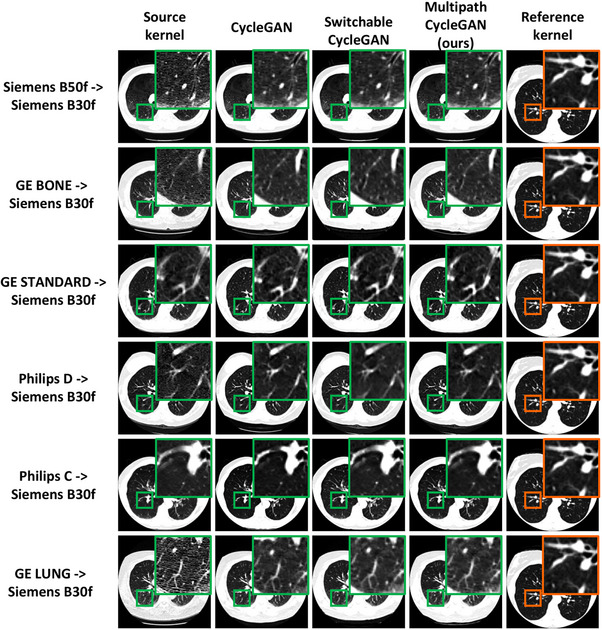
Variation in scanner protocol introduces differences in the texture of the lung parenchyma, thereby introducing differences in quantitative image measures. Harmonization of all kernels to the reference B30f soft kernel enforces consistency in the texture of the lung parenchyma which benefits downstream tasks that include percent emphysema quantification. Multipath cycleGAN enforces consistency in the texture of the lung parenchyma, comparable to baseline models across all unpaired kernels.

Unpaired reconstruction kernels exhibit entirely different anatomical distributions since two different subjects are scanned on two different scanners. This is reflected in Figure 7, depicting anatomical inconsistency and variation in scanner protocols. Our multipath cycleGAN model learns a shared latent space that is capable of harmonizing unpaired kernels to the style of the target soft kernel as seen in the highlighted anatomical region. However, the harmonized GE BONE, GE STANDARD, and GE LUNG kernels show subtle changes in the anatomy for the multipath cycleGAN as compared to the cycleGAN and switchable cycleGAN models. Additionally, the cycleGAN baseline model introduces a shift in the anatomy for the harmonized Philips images.

**FIGURE 8:**
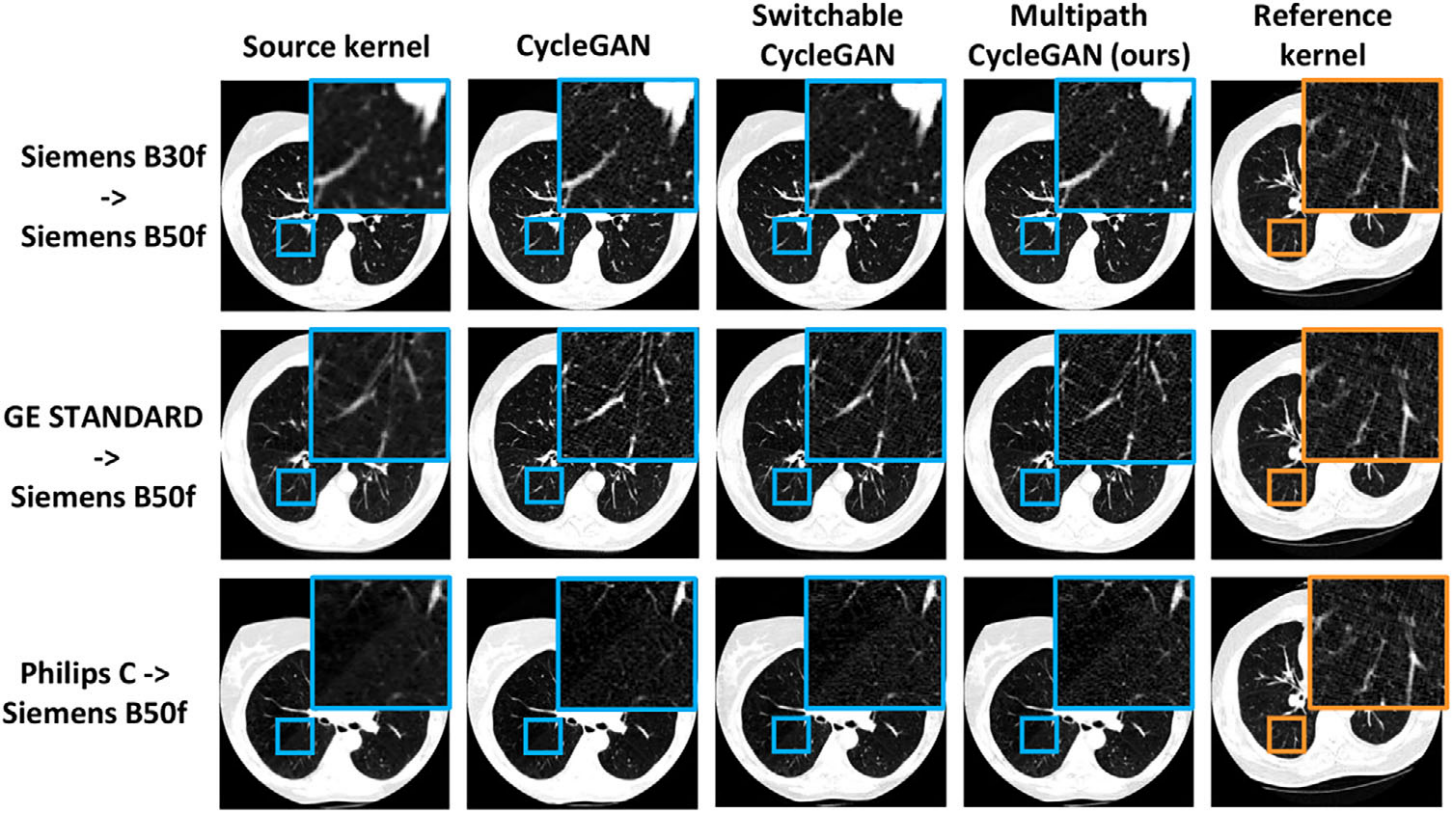
Harmonizing a soft kernel to hard kernel is a challenging task since it is difficult to recover high frequency components from a blurry image. We observe that harmonizing all soft kernels to a reference hard kernel restores the high frequency details in the region of interest in the lungs across the baseline models and our proposed approach. However, the switchable cycleGAN shows a smooth texture for the harmonized B50f kernel.

In the case of harmonization from a soft kernel to a hard kernel for unpaired kernels, it is difficult to restore high frequency pixel noise in a soft kernel image because of variability in scanner protocol. Our multipath cycleGAN model and the standard cycleGAN model restored the high frequency details in the highlighted region of interest seen in Figure 8. The switchable cycleGAN also harmonized the soft kernels to the style of the reference hard kernel. However, on the Siemens kernels, the region of interest is slightly blurry compared to the remaining models.

### 4.5 Impact of kernel on emphysema quantification

We present the results of the general linear model analysis for unpaired reconstruction kernels in Table 3. Prior to harmonization, hard kernels have positive coefficients indicating higher emphysema scores as compared to the reference Siemens B30f soft kernel while soft kernels have negative coefficients indicating lower emphysema scores as compared to the reference. The Siemens B50f, GE BONE, Philips D, and GE LUNG kernels had large positive coefficients while the Philips C and GE STANDARD kernels had negative coefficients. All covariates were significantly different (*p *< 0.05) before harmonization. After harmonization, both the baseline and the multipath cycleGAN model mitigated systematic differences in emphysema scores (*p *> 0.05) for the Siemens B50f, GE BONE, Philips D, and GE LUNG kernels. However, the Philips D kernel continued to remain significant for the multipath cycleGAN after harmonization. While the Philips C and GE LUNG kernels showed an increase in their coefficients after harmonization, they continued to remain significant for baseline models and the multipath cycleGAN. The switchable cycleGAN models eliminated the effects of the Siemens B50f and GE STANDARD kernels. Although the remaining kernels showed an increase or decrease in the coefficients, the effect of kernel on emphysema was partially mitigated and therefore continued to remain significant.

**TABLE 3:** Multivariate linear regression model to assess the impact of age, sex, current smoking status, and kernel on percent emphysema quantification for unpaired kernel harmonization to a reference Siemens soft kernel.

Covariate		Coefficient			*p‐*value			
	Before	After (cycleGAN)	After (Switchable cycleGAN)	After (Multipath cycleGAN)	Before	After (cycleGAN)	After (Switchable cycleGAN)	After (Multipath cycleGAN)
Siemens B50f	11.72	0.58	0.63	−0.07	<0.05	0.35	0.30	0.91
GE BONE	4.13	−1.00	2.51	−1.21	<0.05	0.11	<0.05	0.06
Philips C	−4.00	1.56	−1.70	2.20	<0.05	0.01	0.01	<0.05
Philips D	11.14	0.19	−4.50	2.94	<0.05	0.77	<0.05	<0.05
GE LUNG	12.78	−4.44	−3.93	−2.26	<0.05	<0.05	<0.05	<0.05
GE STANDARD	−5.09	−1.14	−0.40	−0.70	<0.05	0.07	0.51	0.28
Age	0.20	0.18	0.20	0.22	<0.05	<0.05	<0.05	<0.05
Sex	−1.76	−1.83	−2.08	−2.05	<0.05	<0.05	<0.05	<0.05
Current smoking status	−3.18	−2.30	−2.42	−2.68	<0.05	<0.05	<0.05	<0.05

*Note*: We present regression coefficients, standard error, and the *p*−value to explain the impact of each variable on emphysema quantification. After harmonization, we observe that the impact of certain kernels on emphysema is mitigated upon harmonization for either the cycle GAN, multipath cycle GAN, or both models highlighted in bold. The coefficients for age, sex, and current smoking status showed minimal changes and continued to remain post harmonization. Harmonization preserves the biological meaning of the subjects while simultaneously mitigating kernel effects on percent emphysema quantification.

### 4.6 Anatomical consistency after harmonization to a reference soft kernel

**FIGURE 9:**
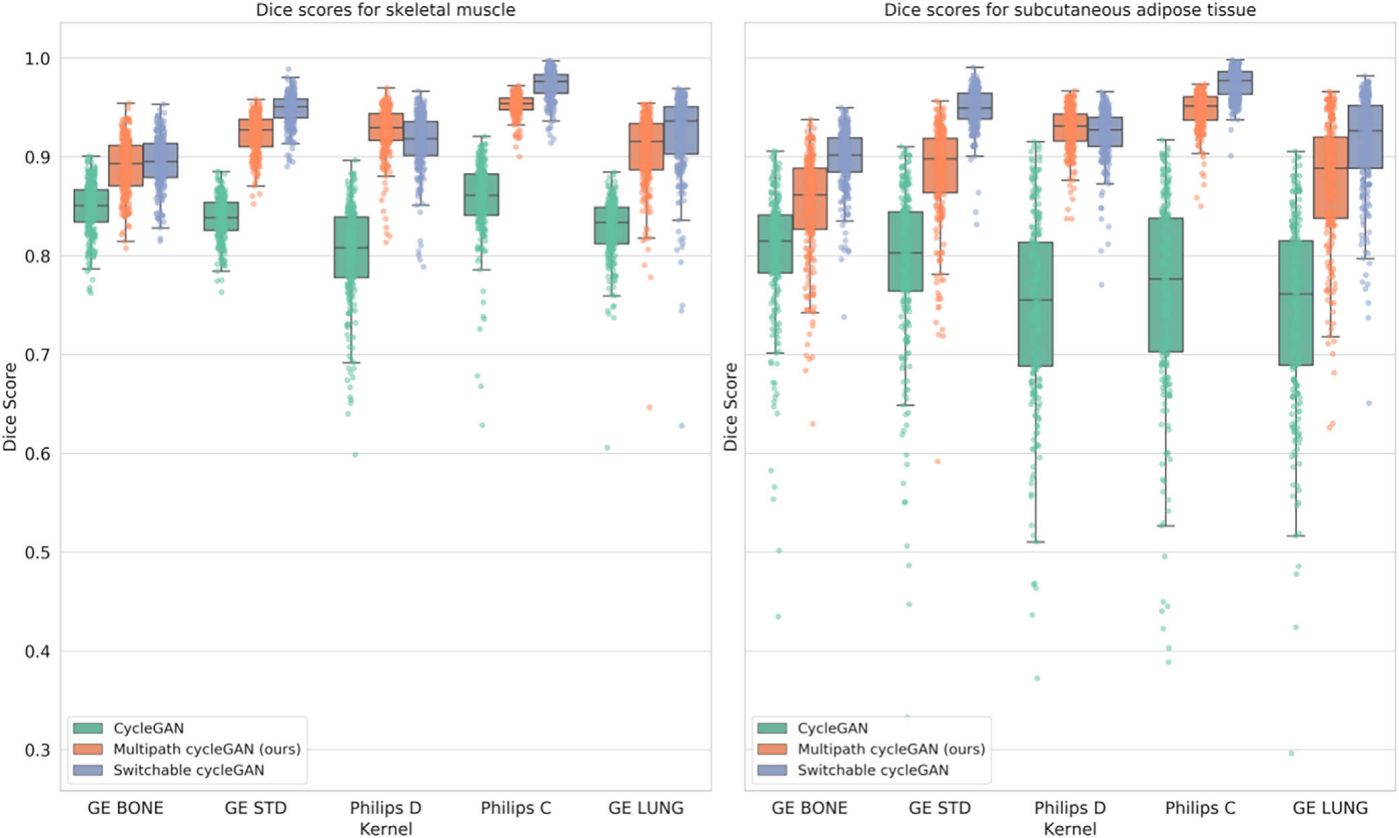
We assess the consistency in skeletal muscle and subcutaneous adipose tissue (SAT) before and after harmonization of the unpaired kernels by computing Dice coefficients between the input source kernel and the harmonized target kernel. The multipath cycleGAN preserves anatomy for all kernels as compared to the cycleGAN for muscle and fat. However, when compared to the switchable cycleGAN model, the multipath cycleGAN model either underperforms or achieves similar Dice scores for muscle and SAT.

We present the Cohen's d effect sizes for skeletal muscle, SAT, and lung vessels in Table 4. Cohen's d was greater than 0.8 for all pairs between Dice on muscle and SAT from the multipath cycleGAN and standard cycleGAN model. The range of Dice scores was higher for all pairs for the multipath cycleGAN compared with pairs for the cycleGAN indicating greater anatomical consistency (Figure 9).

**TABLE 4:** Distribution matching losses in cycleGAN models introduce hallucinations. We quantify hallucinations by measuring the effect size between Dice scores obtained on segmentations of skeletal muscle and subcutaneous adipose tissue (SAT) before and after harmonization using the Cohen's d statistic. Furthermore, we measure the effect size between dice scores obtained on segmentations of lung vessels. Cohen's d quantifies the magnitude of difference between the Dice scores that signifies the degree of hallucinations induced post harmonization. We present the Cohen's d statistic for all unpaired kernels that are harmonized to a reference Siemens B30f soft kernel. The effect size is small when Cohen's d = 0.2, medium when Cohen's d = 0.5 and large when Cohen's d = 0.8. A positive Cohen's d indicated that group one had a higher mean than group two while a negative Cohen's d implied the opposite. Our approach maintains anatomical consistency as compared to the cycleGAN model. However, the switchable cycleGAN model does better on select kernels compared to our approach. For the lung vessels, our approach shows reasonable overlap on most of the kernels compared to the cycleGAN, while the switchable cycleGAN shows good overlap compared to out proposed approach.

	Cohen's d for skeletal muscle		Cohen's d for SAT		Cohen's d for lung vessels	
Harmonized kernel	Multipath cycleGAN (ours) versus cycleGAN	Multipath cycleGAN (ours) versus switchable cycleGAN	Multipath cycleGAN (ours) versus cycleGAN	Multipath cycleGAN (ours) versus switchable cycleGAN	Multipath cycleGAN (ours) versus cycleGAN	Multipath cycleGAN (ours) versus switchable cycleGAN
GE BONE − > Siemens B30f	1.54	−0.15	0.81	−1.08	6.42	0.77
GE STANDARD ‐ > Siemens B30f	3.99	−1.35	1.35	−1.61	24.90	−5.48
Philips D ‐> Siemens B30f	3.12	0.39	2.58	0.20	−0.14	−0.94
Philips C ‐> Siemens B30f	3.34	−1.56	2.54	−1.55	−1.05	−4.81
GE LUNG ‐ > Siemens B30f	2.30	−0.39	1.60	−0.66	7.49	−0.42

When computing the effect size between the Dice scores for muscle and SAT between the multipath cycleGAN and the switchable cycleGAN, the effect sizes were highly variable across all pairs. For the Philips D kernel, the effect size was small. GE BONE showed a small effect size for muscle, while SAT showed a large effect. The negative value indicates that the mean dice for the switchable cycleGAN was higher than the multipath cycleGAN. For all the other pairs, there was a medium effect size for muscle and a large effect size for SAT, indicating that the switchable cycleGAN was able to preserve anatomy much better than the cycleGAN.

**FIGURE 10:**
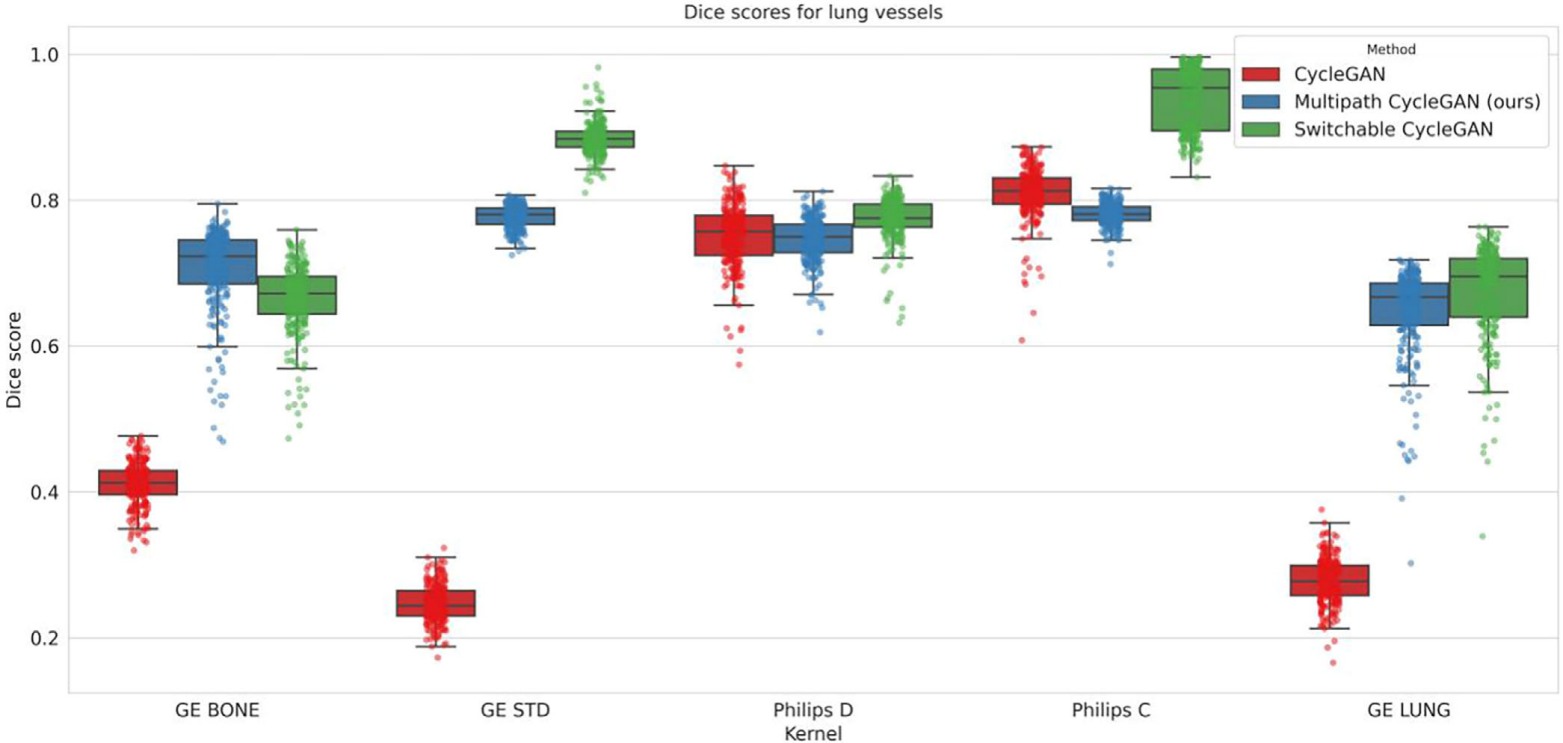
We also assess the consistency in lung vessels and airways before and after harmonization on the unpaired kernels using Dice scores computed between the input source kernel and the harmonized target kernel. The proposed multipath cycleGAN shows reasonable overlap when compared to the cycleGAN model for all the GE kernels, but underperforms on the Philips kernels. When compared to the switchable cycleGAN model, the multipath cycleGAN does better on the GE BONE kernel while underperforming on other kernels.

When compared to the cycleGAN on the lung vessels task, the range of Dice scores for the multipath cycleGAN were higher on the GE BONE, GE STANDARD, and GE LUNG kernels compared to the Philips kernels where the effect sizes were small for Philips D and large for Philips C (Figure 10). The effect sizes computed on the Dice scores between the multipath cycleGAN and the switchable cycleGAN were high on GE STANDARD, Philips D, and Philips C kernels and medium on the GE BONE and GE LUNG images, suggesting that switchable cycleGAN showed better anatomical overlap on most of the kernels.

## 5 DISCUSSION AND CONCLUSION

In this work, we investigate paired and unpaired CT kernel harmonization in a low‐dose lung cancer screening cohort spanning multiple vendors. We develop a two‐stage multipath cycleGAN model with a shared latent space, enabling harmonization across different combinations of paired and unpaired reconstruction kernels within a unified framework. The proposed model is evaluated against cycleGAN and switchable cycleGAN models for percent emphysema quantification and anatomical consistency in unpaired kernels. Additionally, we investigate the impact of kernel on percent emphysema while controlling for age, sex, and smoking status. Our proposed approach mitigates differences in emphysema quantification for paired and unpaired kernels while maintaining anatomical consistency in the harmonized unpaired kernels.

A key component of the study investigates the ability of a shared latent space to enforce consistency in emphysema scores on paired and unpaired reconstruction kernels when compared to separate latent spaces. For paired kernels, we observe that our proposed approach mitigates differences in emphysema measurements post harmonization, consistent with prior works such as Bak et al.,^50^ demonstrating that LDCT scans reconstructed with the Siemens B50f kernel overestimate emphysema compared to Siemens B31f kernel images. For unpaired kernels, harmonizing all kernels to a reference soft kernel reduces variability while preserving the biological signal as evidenced from the coefficient values of age, sex, and smoking status. Additionally, our model can approximately recover the high frequency components when soft kernels are harmonized to a reference hard kernel, achieving comparable performance with baseline models for emphysema quantification. These results suggest that the shared latent space captures features that enable multi‐domain harmonization. However, performance was lower for (B50f, B30f), (C, D), and (LUNG, STANDARD) pairs, as well as the Philips D and Philips C kernels compared to the baseline models. We believe that individual latent spaces can easily learn a one‐to‐one mapping between two domains that can lead to better consistency in quantitative measurements. Nevertheless, domain specific models require separate training for each kernel pair, which is not scalable in large multi‐vendor settings. While our shared latent space results in a performance trade‐off, we can capture a generalizable representation across multiple kernels that enables harmonization in paired and unpaired settings. Our choice of Siemens B30f and B50f as references were based on their impact in lung CT tasks that include percent emphysema quantification.^5^, ^16^ However, our proposed approach can harmonize to any reference kernel that it was trained on, allowing flexibility to choose the appropriate reference kernel based on the downstream task.

When harmonizing unpaired kernels to a reference soft kernel, it is important to ensure that the underlying anatomy remains intact post harmonization. The standard cycleGAN model creates tissue inversion or hallucinations because of the distribution matching losses in the objective function. In contrast, the shared latent space in our proposed approach effectively captures anatomy across all kernels, preserving muscle, SAT, and lung vessels post harmonization. Among the baselines, the switchable cycleGAN achieved the best consistency across all unpaired kernels for all the available anatomy. We believe that the architecture of the switchable cycleGAN involved a polyphase decomposition UNet with skip connections that helped preserve anatomical consistency.

Reliable emphysema quantification is integral to patient care, proving extremely useful in assessing respiratory pathology. Quantitative CT can characterize emphysema patterns, assess severity and evaluate air trapping, improving correlation between imaging findings and histologic patterns.^51^ Assessing air trapping is important for other disease as well, including interstitial lung disease such as hypersensitivity pneumonitis,^52^ and is also an essential finding when evaluating patients following lung transplant.^53^ This analysis can inform management decisions across a range of conditions, from interstitial lung disease to post lung transplant evaluation. Previous studies have shown that inconsistent reconstruction algorithms can lead to differences exceeding 15% in percent emphysema, creating a false appearance of disease progression or improvement in longitudinal follow up.^4^ Furthermore, kernel induced discrepancies can lead to incorrect treatment for subjects undergoing bronchoscopic lung reduction volume. Our harmonization approach standardizes percent emphysema across different kernels, reducing the risk of spurious clinical interpretations, enabling consistent and reliable measurements to monitor disease progression and effectively manage patient care.

Our work is not without its limitations. For the GE BONE and GE STANDARD kernels, while the Dice scores are large for muscle and SAT, our proposed method occasionally hallucinates where muscle and SAT are modified as lung tissues. For this reason, we did not evaluate body composition measures. A potential reason could be attributed to the adversarial training nature that embeds the ability to hallucinate in the shared latent space. This could be mitigated by enforcing additional anatomical constraints through segmentation masks for available anatomical structures. Our current latent space models kernels, and anatomy for the available data. There are multiple acquisition parameters besides kernels that can be added to the shared latent space by explicit conditioning. However, the efficacy of such a latent space requires further exploration. We did not account for slice thickness variability since our proposed model was trained on two dimensional axial slices. We acknowledge the need to account for slice thickness variability through explicit modelling or resampling volumes to a common resolution in future studies. Our harmonization approach focuses on retrospective image harmonization by considering kernels from a specific vendor as the reference. We choose this to ensure a fair comparison across all kernels. The concept of a vendor neutral reconstruction as a target is an interesting idea. However, defining such a target is challenging given the flexibility in kernels that include variations in spatial frequencies and linear optimization. Additionally, in the absence of raw projection data, obtaining a vendor neutral reconstruction target is infeasible. Nevertheless, the flexibility of our shared space could enable the design of novel decoding networks that leverage the properties of this space. This is an area of innovation that requires further exploration. While we show that our model can approximately recover the high frequency components in the lung region during soft to hard kernel harmonization, we acknowledge that spurious data in the form of artifacts could be present in regions beyond the lung, which would require further evaluation. A safer approach in prospective studies would be to store the images reconstructed with hard kernels, allowing for controlled degradation when harmonizing to soft kernels. However, retrospective multi‐centre studies have images reconstructed with either hard or soft kernels.

In conclusion, we developed a two‐stage multipath cycleGAN with a shared latent space for harmonizing paired and unpaired reconstruction kernels across multiple vendors. Our approach mitigates kernel variability in emphysema quantification while preserving anatomical fidelity, ensuring consistent measurements in paired and unpaired settings. With the advancement in CT technology, the need to train domain specific models is burdensome in multi‐centre studies. Our approach provides a unified solution to harmonize paired and unpaired reconstruction kernels that has the potential to advance flexibility, scalability and performance in CT harmonization, thus improving the workflow efficiency in large scale studies.

## CONFLICT OF INTEREST STATEMENT

The authors have no conflict of interest to disclose.

## Supporting information



Supporting Information

## Data Availability

The authors are unable to release the data at this time.
