# Supplementary material for: Multipath cycleGAN for harmonization of paired and unpaired low‐dose lung computed tomography reconstruction kernels
Source: Med Phys. 2025 Nov 8;52(11):e70120. doi: 10.1002/mp.70120 (PMC12596234; doi:10.1002/mp.70120)
Supplement: Supplementary file 1 — Supporting Information [file MP-52-0-s001.pdf]

## Supplementary Figures

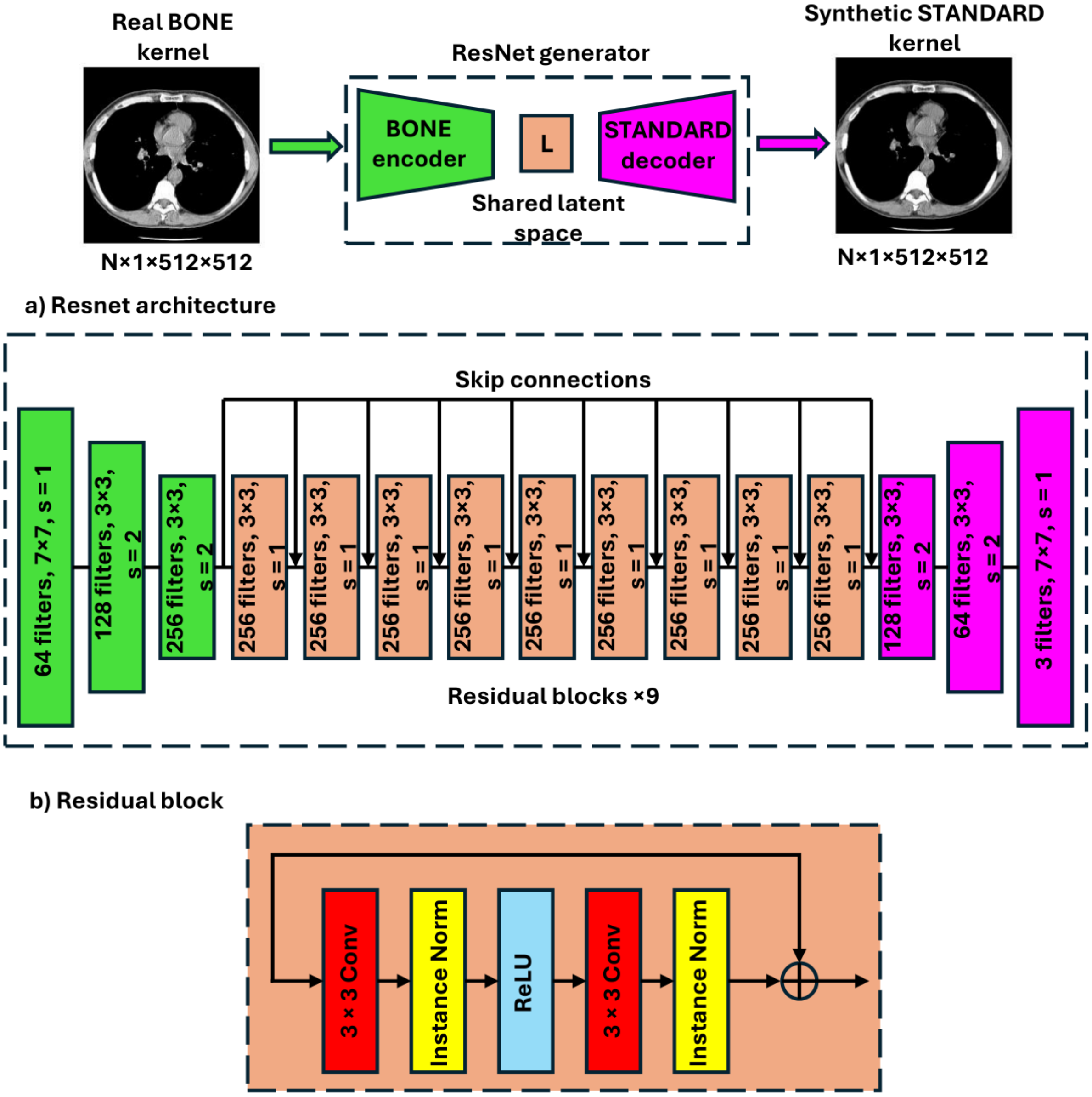


**Figure S1.** The ResNet generator consists of an encoder, shared latent space formed by residual blocks, and a decoder for any given pair of CT reconstruction kernels. **(a)** The encoder consists of three layers with different kernels, filters and strides that progressively downsample the input. The shared latent space is composed of nine residual blocks, each with 256 filters, that preserve the spatial and channel dimensions. The decoder consists of three layers with varying configurations that upsample the feature vector to produce a synthetic image in the target domain. **(b)** Each residual block contains two 3×3 convolution layers with instance normalization and ReLU activation. The input to the first convolution layer is added to the output of the instance normalization layer, resulting in a skip connection.

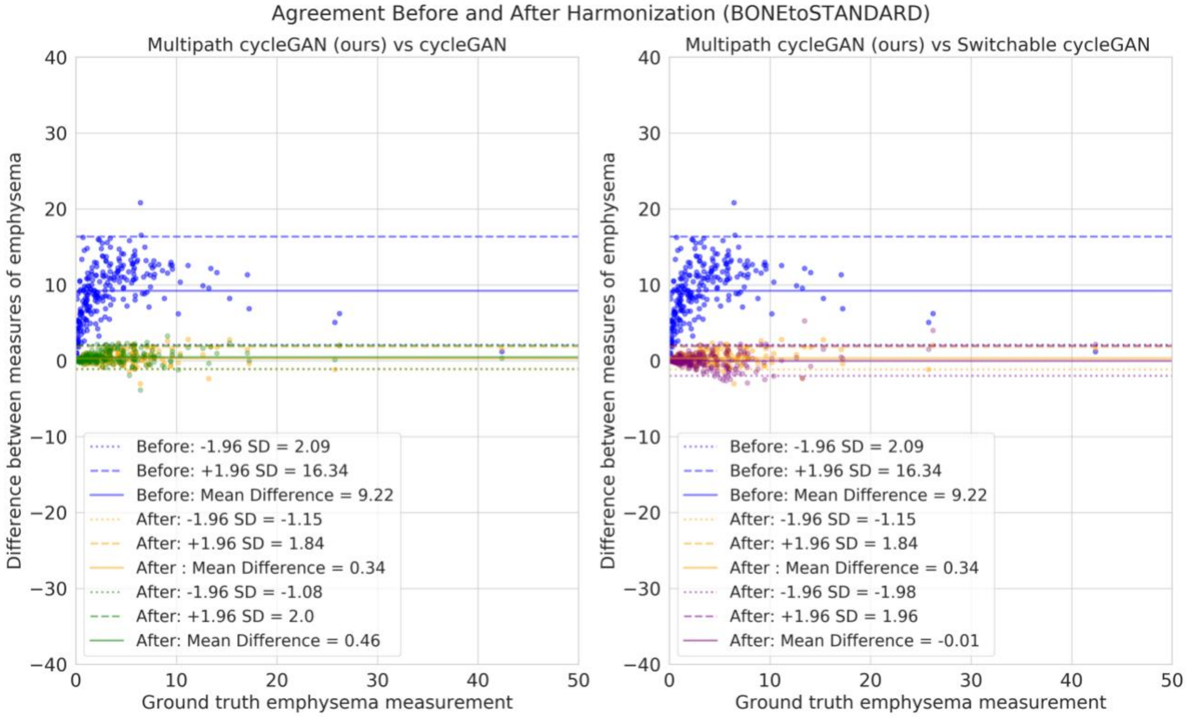


**Figure S2.** Bland Altman style plot for the kernels obtained from the GE manufacturer depicting the performance of multipath cycleGAN versus baseline cycleGAN models. Dashed lines represent confidence intervals, and the solid line represents the mean difference. Blue represents measurements without harmonization, yellow represents the multipath cycleGAN, green represents the standard cycleGAN and purple represents the switchable cycleGAN. Multipath cycleGAN, standard cycleGAN and the switchable cycleGAN model mitigate differences in measurements after harmonization. The multipath cycleGAN achieves a smaller mean difference than the standard cycleGAN while the switchable cycleGAN achieves a mean difference closer to zero.

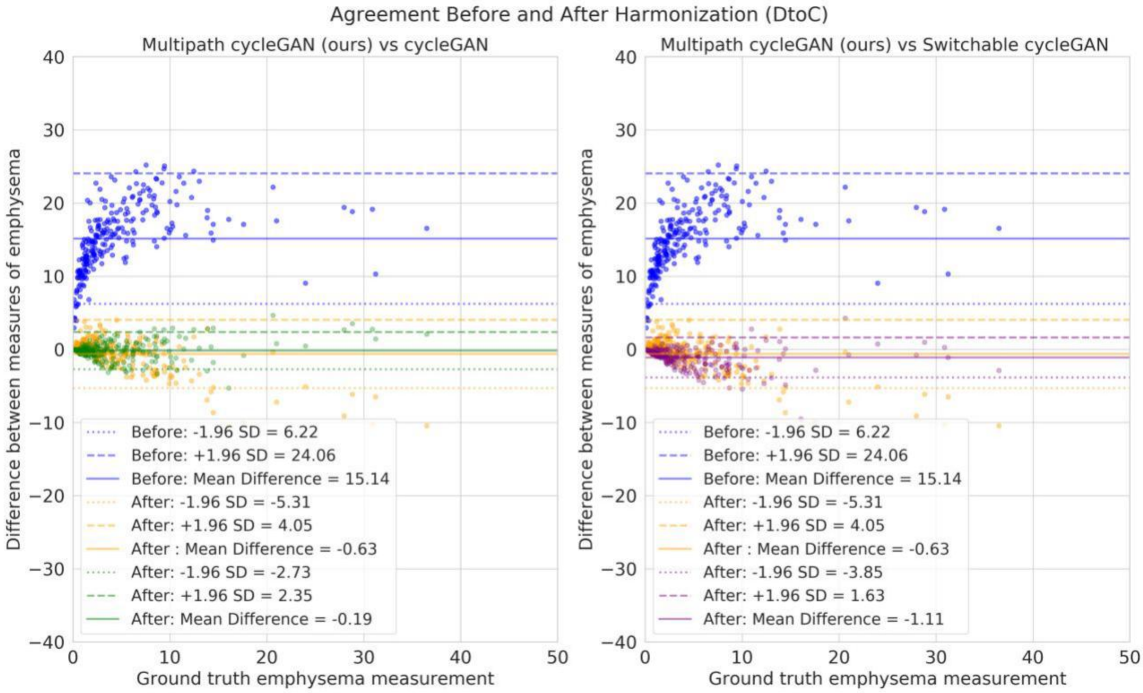


**Figure S3.** Bland Altman style plot for the kernels obtained from the Philips manufacturer depicting the performance of multipath cycleGAN versus baseline cycleGAN models. Dashed lines represent confidence intervals, and the solid line represents the mean difference. Blue represents measurements without harmonization, yellow represents the multipath cycleGAN, green represents the standard cycleGAN and purple represents the switchable cycleGAN. All models mitigate differences in measurements after harmonization. However, the standard cycleGAN and switchable cycleGAN enforce better consistency in measurements compared to the multipath cycleGAN as evidenced by the mean difference and confidence intervals.

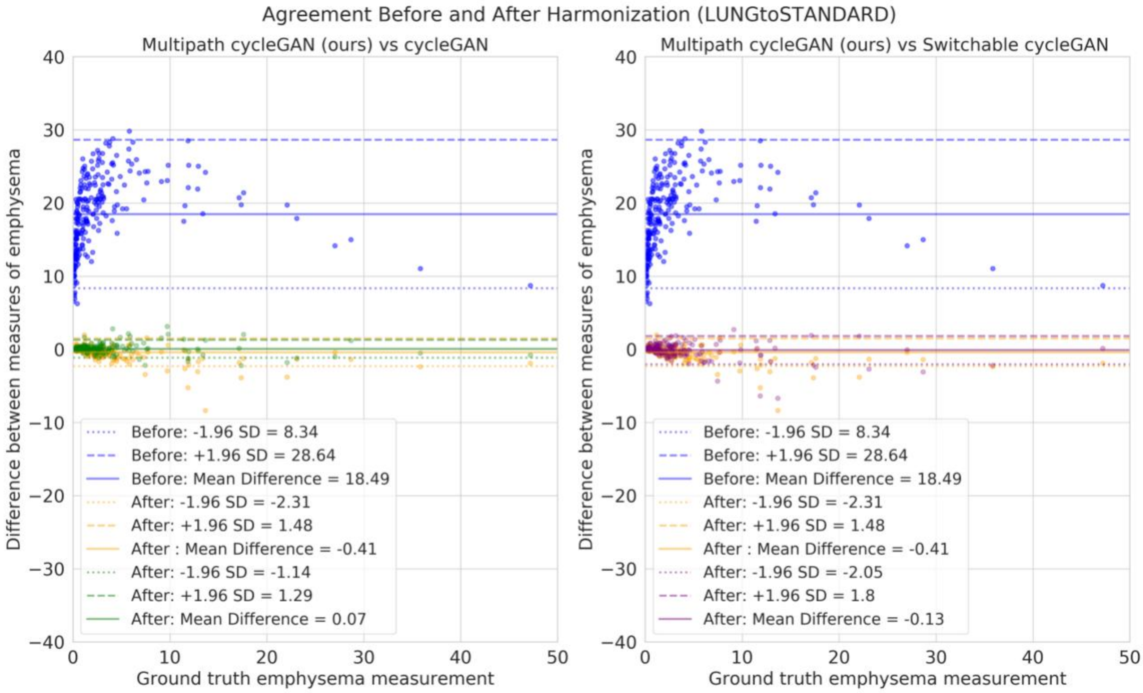


**Figure S4.** Bland Altman style plot for the kernels obtained from the GE manufacturer depicting the performance of multipath cycleGAN versus baseline cycleGAN models. Dashed lines represent confidence intervals, and the solid line represents the mean difference. Blue represents measurements without harmonization, yellow represents the multipath cycleGAN, green represents the standard cycleGAN and purple represents the switchable cycleGAN. Multipath cycleGAN, standard cycleGAN and the switchable cycleGAN model mitigate differences in measurements after harmonization. The standard cycleGAN model is slightly better than the multipath cycleGAN in enforcing consistency in emphysema measurements while the switchable cycleGAN and multipath cycleGAN show comparable performance.

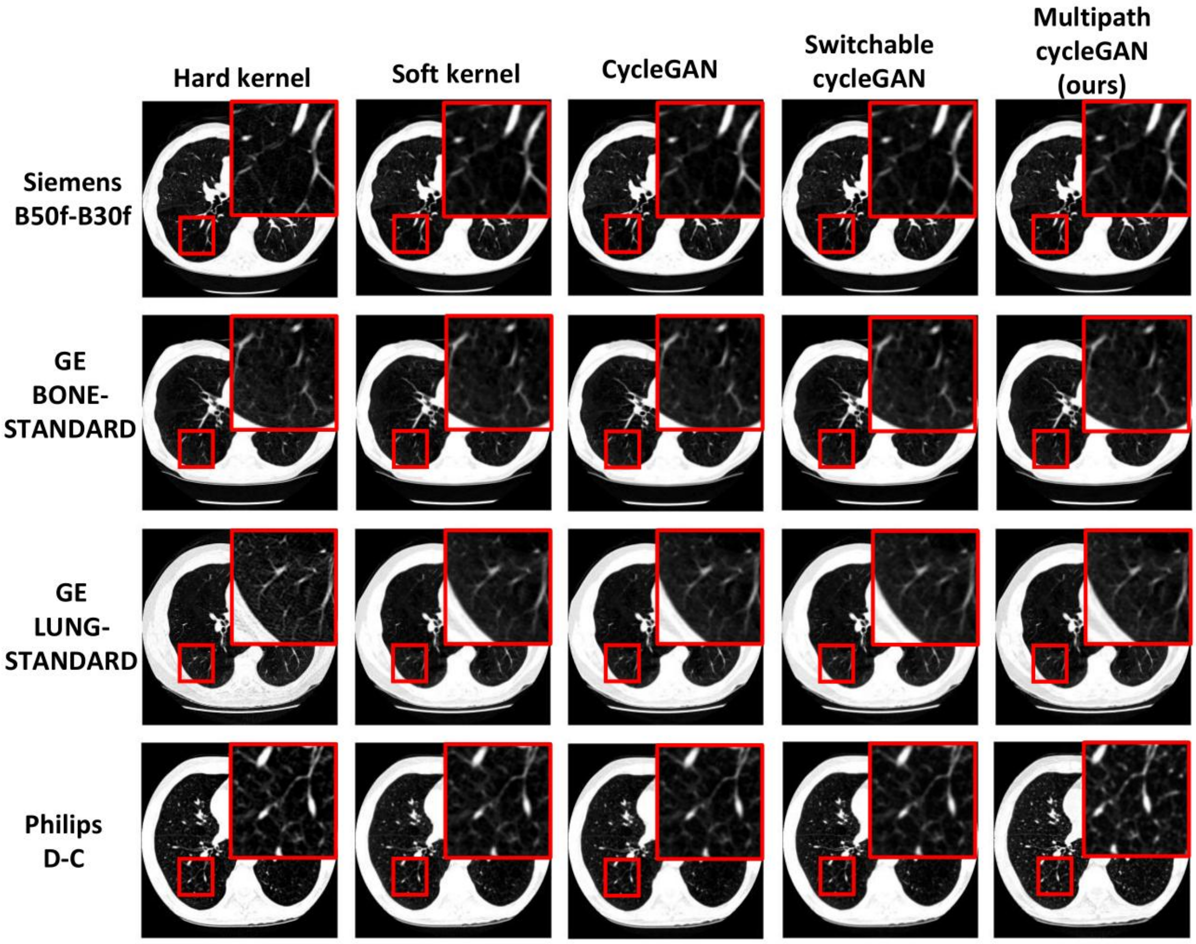


**Figure S5.** We present paired reconstruction kernels before and after harmonization from the 99th percentile of subjects showing severe emphysema. The hard and soft kernels exhibit difference in texture of the lung parenchyma which can impact emphysema quantification. Harmonization enforces consistent texture in the lung, ensuring consistent and comparable emphysema assessment.

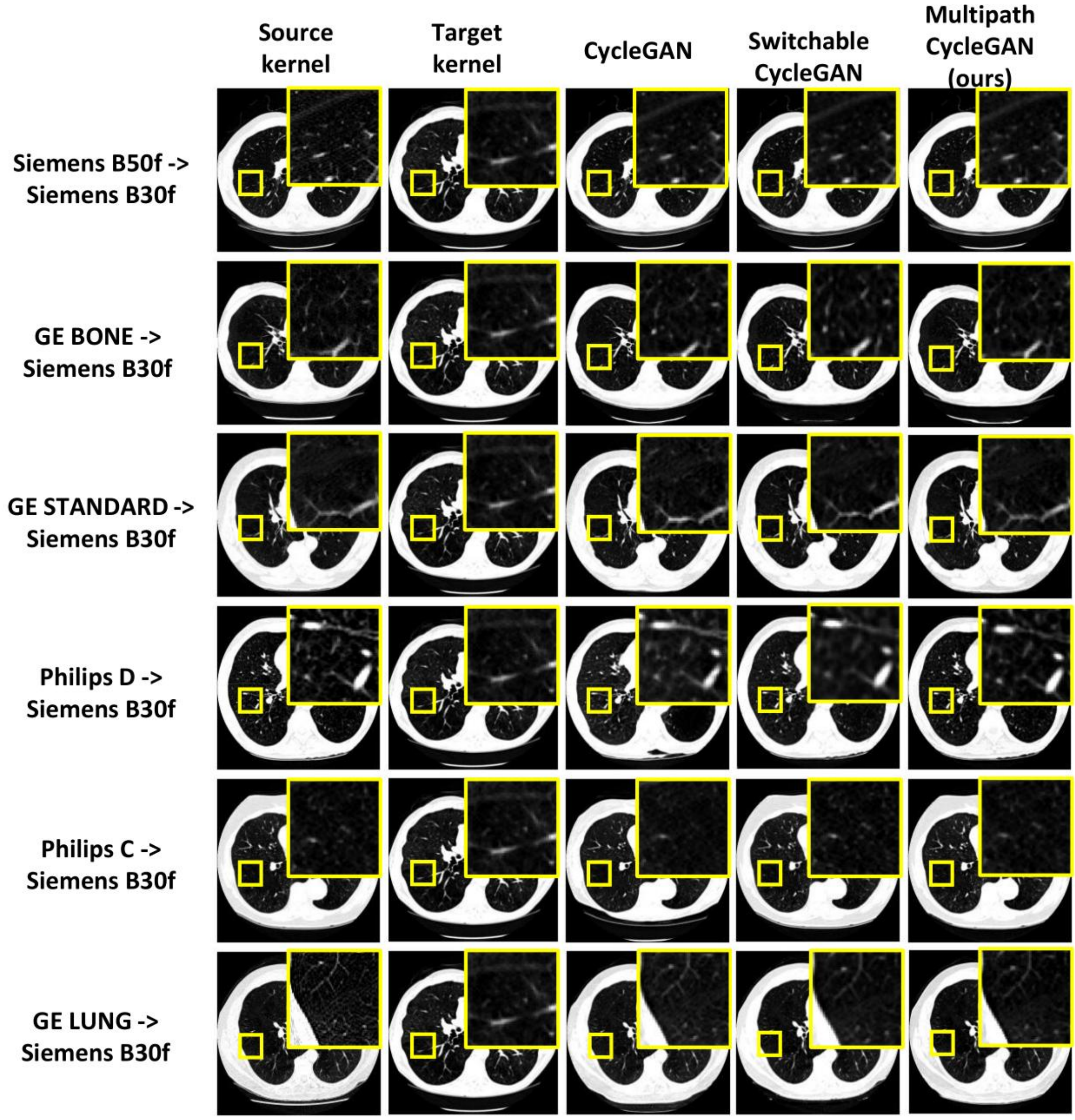


**Figure S6.** We present the subjects from the 99$^{th}$ percentile of emphysema distribution for the unpaired reconstruction kernels. Harmonization of all kernels to the reference soft kernel enforces consistent texture in the regions that show emphysema. However, anatomical hallucinations can be seen on the images harmonized by the cycleGAN and the multipath cycleGAN models.

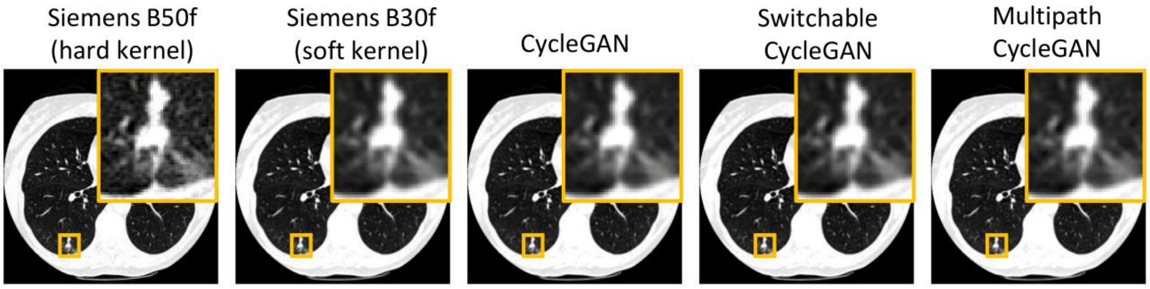


**Figure S7.** The reconstruction kernel impacts the texture of lung nodules. For a given pair of reconstruction kernels from the same subject, the hard kernel (B50f) sharpens the texture of the nodule while the soft kernel (B30f) smoothens it. We observe that harmonization preserves the structural integrity and visibility of the nodule by enforcing consistent texture.

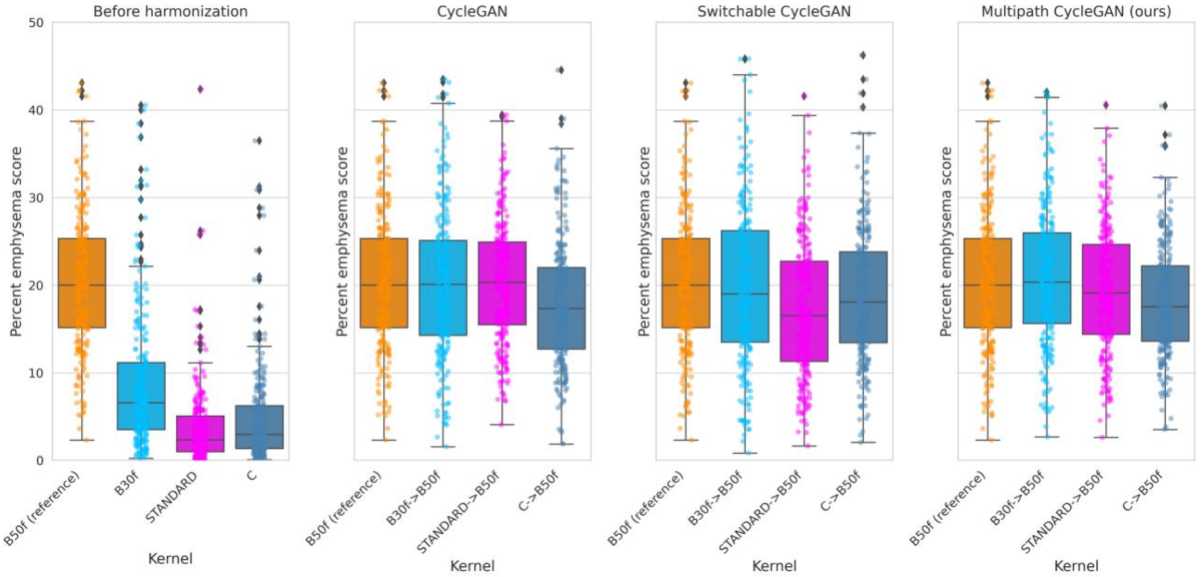


**Figure S8.** Before harmonization, the reference hard kernel, shown as the orange box and strip plot, has a higher range of emphysema scores compared to the soft kernels. Harmonization of all the soft kernels to the reference hard kernel minimizes differences in emphysema measurements We observe that the cycleGAN and our proposed multipath cycleGAN show similar performance with a slight difference in the median. However, the switchable cycleGAN underperforms on all kernels except the Philips C kernel.

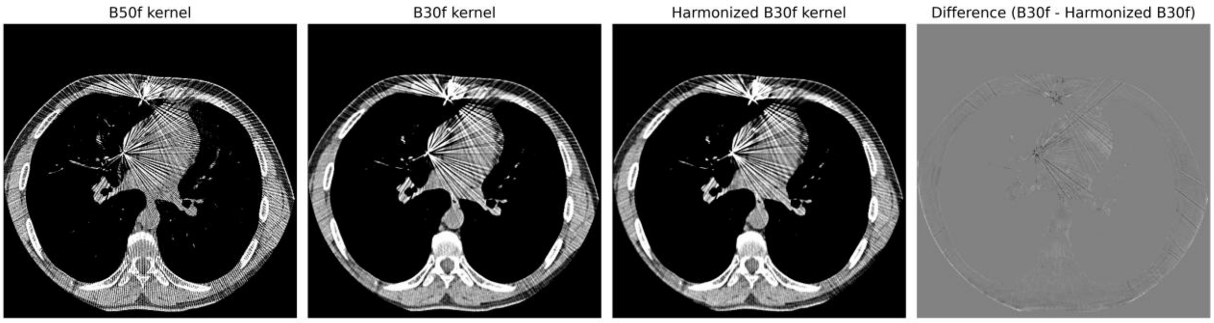


**Figure S9:** Siemens B50f kernel (hard) and the Siemens B30f kernel (soft) show pronounced streaking artifacts when displayed with a window of [-150, 50] Hounsfield units. The harmonized B30f image retains artifacts while matching the style of the ground truth B30f kernel. The difference map shows that anatomical texture is consistent with the reference, while streaking artifacts remain visible.
